# Supplementary material for: “If you find that I am HIV positive, don’t tell me”: Exploring the barriers and recommendations for HIV prevention services utilization among youth in rural southwestern Uganda
Source: PLOS Glob Public Health. 2024 Sep 13;4(9):e0002555. doi: 10.1371/journal.pgph.0002555 (PMC11398690; doi:10.1371/journal.pgph.0002555)
Supplement: S1 File — (DOCX) [file pgph.0002555.s001.docx]

S1 File (Study data -Transcripts, Codebook)

Table of Contents

[FOCUS GROUP DISCUSSIONS 2](#_Toc167405536)

[YOUTH IN SCHOOL FGD1 (YIS FGD1) 2](#_Toc167405537)

[YOUTH IN SCHOOL FGD2 (YIS FGD2) 7](#_Toc167405538)

[YOUTH IN SCHOOL FGD 3 (YIS FGD3) 11](#_Toc167405539)

[YOUTH OUT OF SCHOOL FGD1 (YOS FGD1) 15](#_Toc167405540)

[YOUTH OUT OF SCHOOL FGD2 (YOS FGD2) 18](#_Toc167405541)

[YOUTH OUT OF SCHOOL FGD3 (YOS FGD3) 19](#_Toc167405542)

[KEY INFORMANT INTERVIEWS 22](#_Toc167405543)

[KEY INFORMANT INTERVIEW1_DISTRCT HEALTH OFFICER (KI11 DHO) 22](#_Toc167405544)

[KEY INFORMANT INTERVIEW2_DISTRICT COMMUNITY DEVELOPMENT OFFICER (KII2 DCDO) 23](#_Toc167405545)

[KEY INFORMANT INTERVIEW3_ DISTRICT EDUCATION OFFICER (KII3) 26](#_Toc167405546)

[KEY INFORMANT INTERVIEW4_DISTRICT FOCAL PERSON IN CHARGE OF HIV/AIDS (KII4) 28](#_Toc167405547)

[IN-DEPTH INTERVIEWS 30](#_Toc167405548)

[INDEPTH INTERVIEW NUMBER1_HEALTH WORKER (IDI_HW01) 30](#_Toc167405549)

[INDEPTH INTERVIEW NUMBER TWO_HEALTH WORKER (IDI HW02) 33](#_Toc167405550)

[INDEPTH INTERVIEW THREE_HEALTH WORKERS (IDI HW03) 34](#_Toc167405551)

[INDEPTH INTERVIEW NUMBER ONE_VHT (IDI VHT01) 37](#_Toc167405552)

[INDEPTH INTERVIEW NUMBER TWO VHT (IDI VHT02) 38](#_Toc167405553)

[INDEPTH INTERVIEW NUMBER THREE VHT (IDI VHT03) 40](#_Toc167405554)

[INDEPTH INTERVIEW ONE_TEACHER (IDI T01) 42](#_Toc167405555)

[INDEPTH INTERVIEW TWO_TEACHER (IDI T02) 45](#_Toc167405556)

[INDEPTH INTERVIEW THREE_TEACHER (IDI T03) 47](#_Toc167405557)

[CODEBOOK 50](#_Toc167405558)

[Theme: Recommendations to utilization of HIV preventive services 54](#_Toc167405559)

[Summary of themes and sub-themes 55](#_Toc167405560)

[Summary of category and codes 56](#_Toc167405561)

# FOCUS GROUP DISCUSSIONS

## YOUTH IN SCHOOL FGD1 (YIS FGD1)

**Qun1.** **Can you describe a typical day in your life?**

**Answers**

**Speaker Number Eight (8**): I am number 8. it was October 2021 when unfortunately lost an OG just because of HIV that at times spread though playing with sharp instruments , sexual intercourse or through accidents .yeah…it was so unfortunate though it was due to may be her carelessness but of what was not except during Colona Virus many youth those who were HIV positives got some challenges where you could find someone who was taking her tabs far away from her district because of self-neglecting and whatever but when Colona came ..,you could find that you have to get the ARVS from the home area where you could have abused or either not discussed about very well with the patient of what should have been used so my friend Auge just got self-neglecting her life also was just neglecting herself … kale abusing her life because of having HIV/AIDS of which was not good because she got it by birth . Therefore, due to luck of parental care and someone to advise her to take her tabs and whatever, unfortunately died due to HIV in October 2021.

**Speaker Number four (4):** I am number four and the day I experienced such a terrible thing was the day! when my friend tested HIV positive .it was due to playing sex with those prostitutes and on testing, he was tested positive and after going home due to pressure and fear of his parents, he killed himself.

**Speaker Number one (1**): I am number one..., the typical day that I experienced was in March 2019 my aunt tested HIV positive. It was really a tough time for her to recover since HIV Can’t be recovered so my aunt could feel …. very unlucky like such unlucky person that she cannot do even anything, and she cannot even go in the community with us…, she could just be alone and just feel neglected at some times and with that, she hanged herself. it was really a tough day for us. That what I can say.

**Speaker number six (6**): I am number six, it was last year on Augst when we got an accident and the vehicle where we were in... some people were HIV positive but when we went on testing after an accident, some people whom I were siting with tested positive but me never tested positive. I just thank God for that. That is what is all what I have.

**Speaker Number two (2):** I am number two, the only uncomfortable experience I faced in the long vacation is that many girls got AIDs because they loved money and most people with money where victims and so they were also made to have AIDS**.**

**Qun 2.** **What do you know about HIV/AIDS?**

**Answers**

**Speaker Number two (2):** I am number two, what I know about HIV is that it has no medicine, once it attacks you, you don’t get healed and the way of preventing it is abstinence, use of condoms and…...

**Probe by Justine.** How do those ways of preventing it affect the youth?

**Speaker Number four:** But some girls…. need money and they try to go to people with money ‘the men’ and you may find a man having money since he is a victim and when you go to get money, you finally get AIDS through sex.

**Speaker Number Eight:** yeah, at times **… I** am number eight and the ways of abstaining **or** the ways of preventing HIV, yeah…. Its right to abstain from sex but…at times to make generation after generation it’s needed as it’s a reproductive part of the life because you need to generate from one generation to another so that people can continue to exist yes you can’t know that one has HIV by face, no. but at least one can take care of herself and reach a level ,some work or an age where you can find the right partner yeah even though you find the right partner of your heart has HIV ,you may find the way of living together and no problem can occur . You may have AIDS and I don’t have it, but we live, and the children of our generation also lives. Then another thing of using condoms and whatever, also its dangerous though we emphasised to use them at times condoms you may use them hidden but not educated very well on how to use them most especially senior classes and primary level classes like P.7, P.6 and P.5 though we think that they know nothing about sex and whatever, but. seriously they are mostly affected by HIV because they don’t have care from their parents because they are always busy, no care from teachers because they fear them and from the elders so that’s why I am saying that the use of condoms at some times its dangerous because you may find instantly or carelessly and you find that it has gone into the vagina or the truck then at times your forced to go to the hospital to remove it or your find that your forced to get cancer .So that what I have .

**Qun 3. What challenges do youth living with HIV/AIDS face?**

**Speaker Number one (1**): I am number one now to the challenges those people do face most of them we normally provoke them because you can’t be living with HIV and then ….it depends on my character I may provoke you ,insult you and another thing is that they are hated in disguise .I may not show to you that I hate you in real life but then when I hate you …..Another thing we don’t normally share with those people that do have HIV/AIDS due to fear that I may also get infected though they say that to get HIV is through blood ,but you might find that me I do fear to even use the fork that you have used because you have HIV /AIDS and I don’t have it. That what I can say.

**Speaker Number Eight (8):** I am number eight and the challenge that are faced by those students living with HIV especially those in schools, is the mode of distribution of ARVs since they are not provided by the school administrators. That what I can add on.

**Probe on poverty as a challenge to HIV patients**:

**Speaker Number four (4):** I am number four, the people who have HIV being poor affects them in this way; HIV patients need to feed well so that they can get energy then you may find that a poor person does not have enough money to buy food. That what I can say

**Qun 4. In what ways do you think you can protect yourself from getting HIV?**

**Speaker Number six (6):** Number six, we can protect him/herself from HIV/AIDS through abstinence and may be by use of condoms also and other one is ……

**Speaker Number Eight (8):** Number eight, one can prevent from getting HIV/AIDS at least through massive education, TVs ,radios and some guidance from our parents who are somehow good the another one is at least government should provide some ways or some sectors to improve on the way of life of our parents most those who are poor that cannot cater for their children because mostly AIDS is caused by poverty in our homes where you find that I am not provided with anything when I am coming to school so I will be forced to find a sugar dad who can provide me with something yet I don’t know if he is positive or negative so at least the government should find ways on how to cater for children born in backward families or find other ways.

**Speaker Number two (2**): I want to supplement on speaker number 8, also AIDS can be prevented through avoiding peer groups because if you befriend someone or a peer group likes escaping, attending bars so you may also meet some challenges. That what I can say.

**Speaker Number one (1):** I am number one, AIDS can also be spread when those parents of ours or our teachers do not provide us with counselling services because you may find that I need counselling to stop something. Also, too much leisure time you may find that I go with my go with my friends to party or to other places and those places are many boys who might influence me to get Aids. Thank you.

**Qun5. In what ways do you think youth out of school get knowledge on HIV prevention strategies**?

**Speaker Number six (6):** Number six, they get knowledge through watching Television**.**

**Speaker Number (8):** Number eight ,youth out of schools especially those ones deep in the villages may at least get knowledge on how to prevent HIV one from massive education via radios since it’s the quickest means of communication in Uganda because in 2019 when we had COVID 19,the government emphasized that at least everyone to have a radio so they may be emphasized or guided through radios .Then another one may be the government through local councils ,groups ,churches may emphasize parents to take care of their children not only girls but also boys.

**Qun6. Tell me in which situations one may be prompted to test for HIV**

**Speaker Number two (2):** Number two, one may be required to test for HIV especially after playing sex with someone he /she doesn’t trust**.**

**Speaker Number four (4):** Number four, some may test for HIV when the government extends testing services to the health facilities such as monthly testing of HIV.

**Speaker number eight (8):** Number eight, one may go for HIV testing when you have got an accident either in a tax or a bus.

**Qun 8. Tell me why you think some youths do not test for HIV**.

**Speaker number one (1):** The reason why some youth deep in the village don’t test for HIV is that some of the are very proud of themselves. They just say that me to acquire HIV! No…they just have that feeling. Then another reason why some youths don’t go to test for HIV is because of peer group influence. You’re my friend then I ask you why you go to test for HIV? there is nothing long with you and you can’t go to test for HIV. Thank you.

**Speaker Number Eight (8):** yeah, that is the challenge that have happened to me inclusive there is a way how nurses and doctors at the health centers do act one is that you go to test for HIV and your then forced to transmit the blood’ blood transfusion ‘ so that they get your blood and when they test and they find that your HIV negative , they then use your blood for other purposes like me I can’t go to test for HIV yet they need like one litter or a half of blood .

**Speaker Number two (2):** some youths don’t go to test for HIV because workers there at the health Centers when you go to test and you find that your HIV positive, they spread rumors that this one is positive, hence affecting you.

**Qun 7. What do you think is the next step after one has tested positive for HIV?**

**Speaker Number four (4):** As they did in Colona because people were forced to test before crossing any place. That law should also be implemented in the HIV sector so that someone to go somewhere first tests for HIV. That one will be easy for someone to test.

**Speaker Number (8):** At least the health workers in the government hospitals and health centers should be advised to speak politely to the patients.

**Speaker Number one (1**): In my opinion, I do think that people like let me give an example like MC Kats tested positive then she informed the whole world that she is HIV positive so those people like MC Kats should be used to counsel those people with HIV or Negative such that they become much aware of what is going on. Thank you**.**

**Speaker Number four (4):** Then…ways of encouraging people to test, there is another one-educating people about dangers of staying with HIV.

**Probe on how long a person living with HIV should take on treatment by Justine**

**Speaker Number eight (8):** Before we proceed, I have a question on that one. Is ARVs the only medication of HIV or there are other injections used or syrups just want to know about that…….

**Response by RAs:** per now ARVS are the only medication used though they are trying to introduce an injection but that one is not yet published no where so we are not sure that there is an injection of HIV, we are now focused on ARVs because that is what we have. [speaker number 8]: Then what of blood transfusion? [response]; we are also not sure about that but what we know is ARVs.

**Probe** .do you think that ARVs cure AIV/AIDS according to your thinking?

**Speaker Number eight (8**): yeah…according to me, ARVs do not cure HIV/AIDS but they prolong the gestation period for you on earth so that you can still be seen by your beloved ones, but they do not cure HIV

**Qun 8. How best do you think we improve access to the above HIV/AIDS care and treatment especially for those youth living with HIV/AIDS in school?**

**Speaker Number four (4):** on that issue, the government should provide medication in schools such that when someone is having HIV, it becomes easy for him /her to approach the administration and take the drugs than going home.

**Speaker Number Eight (8):** on how to take ARVS for the prevention of HIV in schools, yeah…. they may provide them at school administration but on what conditions? I am a student with HIV at school and the students never knew that I am HIV positive, the students will see me picking drugs at the school clinic! Therefore, they will take me as if I am a student not of their character or whatever. Some will not go there for them.

Then another thing it concerning with private schools at times we are not provided with a lot of drinks and fine food for the ARVs patients, so they get a challenge when taking their drugs. Either the government should provide at least in some government schools where the HIV patients could study from where there are some appropriate medications and food provided to them. Thank you

**Speaker Number one (1):** In my opinion, I do suggest that those students that test HIV positive should be placed in one school and taken care of.

**Qun 9. What could facilitate better utilization of HIV/AIDS care, treatment, and prevention services for the youth out of school?**

**Speaker Number two (2):** For me I think you find for those people in the deep village in the all-sub county, they have like one health center/hospital, and this becomes difficult to get services. So, the government should at least extend health centers /hospitals to parish level such that when one is sick, he can easily approach the hospital. Thank you

**Speaker Number eight (8):** how the non-going school could acquire HIV/AIDs care ,treatment and prevention services ,yes the health centers and hospitals are there but at times you find that the tabs are done and when you go to the clinics or hospitals ,they tell you that there are no drugs which is also happening to the Colona virus injection because when you get the first injection and go there for the other injection ,they tell you that it is not there and this is what happens to the HIV/AIDs patients when he/she goes there, they tell him that there is no drugs and he finishes the whole week minus taking the drugs and when you fall down and die, they say that HIV has killed someone yet it is the carelessness of the government to provide essentials in the hospitals.

**Qun10. Is there something you want to add to this interview, something we didn’t talk about but that you feel is important to you and want to share?**

**Speaker Number one (1):** yeah…you didn’t ask for those HIV patients on how to care about them

**Probe: In your opinion is there something that you would like to share to us so that we can take it as a note because that is a good question?**

**Speaker Number one (1):** Yeah…. the government should put strict laws on those people who provoke those suffering from HIV such that when those people are caught, they should be able to face the law**.**

**Speaker number five (5**): I would like to disagree with that one who has said that we should build schools for those infected from HIV suggesting that this should not be done because they discriminate them from others which is not good**.**

**Speaker Number eight (8) :**I disagree with the one that has said that the government should put strict laws to those people that provoke the HIV patients suggesting that this will increase the incidents of HIV since HIV patients will be seen as people at the highest level than others .yeah…you will find that when I have HIV,I am not talked about because once you talk about me, you will be in the courts of laws. So, I will be encouraged to get it so that at least be on High level. At least the government should find ways like when you go at the health center to pick ARVs go with your parent /guardian who take care of you. Yes, at times they take ARVs at home and just take them for decoration and at least if someone go with the parent /guardian even tough your mad, he can help you to take them. I think that one can work better but not giving them many rights. Thank you.

**Speaker number three (3):** In my opinion of what we have talked about, I think the government should encourage people to grow vegetables to those people that are poor because they are more nutritious in them. That is all that I have.

**Qun11. Are there any questions you have for the researcher?**

**Speaker Number Eight (8):** yes, we have talked about how AIDs are spread, but mostly it is spread through rapes. yeah…. girls of young age or the teenagers are raped sometimes. Yes, others are raped, and others acquire it from their wish.

**Probe: They are raped? how**

**Speaker Number (8):** Yeah, some are raped when they are moving on their way by rapists while others are raped as a result of violence and disagreements from their neighbors hence hiring someone to rape them because of enmity and others are being infected by their step mums by injecting them with an infected blood especially the young ones because of jealousy.

And other thing we should educate HIV patients on how to take care of their blood because you can meet it anywhere and you don’t know if it was from an HIV patient, yet you also have an injury and infects you live with it unknowingly.

**Speaker Number seven (7):** I want to supplement on the one that has said that HIV patients should be separated because that will be a very good thing since those youth in schools who are not infected will also protect themselves from getting it so that they don’t join such schools but if we remain mixed, for sure you cannot know who is infected and who is not and you cannot prevent yourself from dating because that is normal.

## YOUTH IN SCHOOL FGD2 (YIS FGD2)

**QUN1:** **Can you describe a typical day in your life?**

**Answers**

**Speaker Number Three (3):** I have my aunt at home and she is living with HIV/AIDS, the problem she has is that she can’t take ARVS on her own without forcing her and another problem she has is that she fears to go to the health centre to pick her ARVS. She also drinks alcohol without even eating which makes her body to remain in a danger. This is why you find that most people who are on ARVS die abruptly without knowing the cause as to why they have died in such away.

**Speaker Number one (1):** The most problem people living with ARVS have is poor feeding where by you find someone on ARVS eating poorly yet they should be eating well. Therefor most times, poor feeding is the major cause of death in such people.

**Speaker Number seven (7):**For me I still remember like a one year during COVID I had my friend and she was having her boyfriend and one day they decided to go into outing, they went there…,they have never gone for testing and they did whatever they did…and when they returned, she got some challenges ….where we find she was not feeling well ,feeling headache like for almost a month after I told her and I advised her that why can’t you go for testing she refused telling me that if you were you, you can fear. After I forced her and went with her in the hospital, and we found that she had an HIV. After she started…. she refused even take tablets and she ended up dying………

**Qn 2. What do you know about HIV/AIDS?**

**Speaker Number Three (3):** As number three, HIV is a disease that has no what! No cure and even that disease if you’re not treated, you die but if your treated, you will be reducing the high speed of it. As me number three, that how I know that HIV is a dangerous disease and for sure, I encourage those young youth like me! To stop playing sex with other students, their neighbors in order to avoid that disease because that disease is very dangerous even it destroys your future if you’re not serious**.**

**Speaker number seven (7):** For me I know that HIV is a virus that spreads AIDS, and that disease has no cure only that you can get tablets to keep on reducing it, but it can’t be prevented.

**Speaker Number Eight (8**): For me I think HIV is a disease that is spread through sexual intercourse and using sharp objects with an infected person**.**

**Speaker Number six (6):** For me I think HIV is a disease that is spread through sexual intercourse and can’t be cured.

**Probe: which is the commonest mode of spread of HIV in your community?**

**Speaker Number Six (6):** HIV is spread through sex, even on sharing sharp objects.

**Speaker Number (1):** According to my thinking, HIV is mostly commonly spread in schools through homosexuality even students play sex in the schools without using condoms and even sharing sharp objects. That’s what I can say.

**Speaker Number eight (8):** The major cause of HIV in schools is that you find someone with HIV but dating someone without HIV and not openly telling him or her that he/she has HIV positive. This results into easy spread of HIV in schools because even one person can infect the all school.

**Qn3. What challenges do youth living with HIV/AIDS face?**

**Speaker Number seven (7):** Me as number seven I think you may end up neglecting yourself and end up killing yourself.

**Speaker Number one (1):** There is school neglect after knowing that your HIV positive, even friends fear you after knowing that your HIV positive and even fear to sit near you in class and even sharing with your materials like forks, plates, thinking that you may infect them through such materials.

**Speaker Number two (2):** Those students who are HIV positive, they even lost some weight. You see a patient you have HIV not even having power to work for the future.

**Probe on how poverty is a challenge on HIV patients**

**Speaker Number Three (3):** Yes, me as number three poor can be, I mean poverty can be a challenge to that person because if a person with HIV and you don’t have money to buy food, to feed on for sure you end up dying because without food, you cannot live.

**Speaker Number six (6):** You find someone with HIV/AIDS lacking transport to go to the health center to pick tablets because of poverty.

**Speaker Number five (5):** someone with AIDS should be eating well, but because of poverty, they lack money to buy good food which results into death and showing the signs of HIV at an early stage to the public which rises stigma among HIV patients.

**Qn 4. What solutions would you suggest tackling the above challenges?**

**Speaker Number (1**): Head teachers should try all means to fight the habit of coupling at their schools and if possible, provide condoms at their schools from hospitals for those students who can’t stop having sex.

**Speaker Number (3**): The government should try by all means to build health centers nearer to the people in the community and avail ARVS to them so that those people who move long distances to get these services finds it easy to get them nearer.

The government should also try by all means to bring condoms and distribute them in every home so that everyone knows how to use condoms in order to protect themselves from HIV/AIDS.

**Speaker Number five (5**). I advise my fellow students to abstain from sex until the real time or if they can’t abstain from sex, they should use condoms.

**Qun 5. In what ways do you think you can protect yourself from getting HIV?**

**Speaker Number Eight (8):** By the use of condoms**.**

**Speaker Number one (1):** Avoid sharing sharp objects**.**

**Speaker Number Three (3):** Avoid coupling with a person which is affected by HIV**.**

**Speaker Number Five (5):** You can abstain from sex and be faithful to your partner**.**

**Probe on the sex education, counseling services and laws that protect students living with HIV/AIDS.**

**Speaker Number Three (3):** For me I can’t lie, we don’t have those laws in our school and counseling services.

**Qn 6. In what ways do you think youth out of school get knowledge on HIV prevention strategies?**

**Speaker Number Three (3):** I think they get such information from village meetings especially from VHTS.

**Speaker Number seven (7):** I think they should introduce guidance and counseling services on how to prevent HIV in villages.

**Qn 7: Tell me in which situations one may be prompted to test for HIV**

**Speaker Number (7):** you may find that me as a girl I have played sex with a boy, and I don’t know whether he is positive or negative then after I am not feeling well then, I have to go and test.

**Speaker Number Three (3**): when you use a sharp object like a razor blade and cut yourself which is mostly been used by someone infected with HIV/AIDS in your home or school.

**Speaker Number six (6):** I suggest if you have your girlfriend, before you start sexual intercourse with her, you should first test for HIV/AIDS to know your status**.**

**Speaker Number Eight (8**): For me I suggest that we should always go for HIV testing to know our status even though we don’t play sex.

**Prejudice by speaker number two.** Is it true that if you drink a Merinda soda and go for HIV testing even if you have HIV, they can’t recognize it.

**Qn 8.** **Tell me why you think some youths do not test for HIV.**

**Speaker Number one (1):** Most of the youth fear to test for HIV because they fear to take ARVS in case they find that they are living with HIV. While others fear to take ARVS in public so they don’t want people to know that they are living with HIV/AIDS.

**Speaker Number five (5):** Majority of the youth take AIDS for granted and by the time they find that they have HIV, it rises stigma in them and fear to take tablets.

**Probe on what should be done to tackle these challenges of HIV treatment**

**Speaker number Two (2):** My suggestion is that government should construct schools of those youth living with HIV like how it constructs schools of those who are needy so that they can take they tablets without fear because when we are mixed with them, they fear to take their tablets in time for the fear of being seen by their fellow students.

**Speaker number three (3)**: The government should come in schools and test students so that if they find some students with HIV, they start they tablets immediately and the school should be responsible for those students. This will minimize the spread of HIV in schools.

**Speaker number six (6**): Government should sensitize those who are suffering from HIV.

**Qn 9. How best do you think we improve access to the above HIV/AIDS care and treatment especially for those youth living with HIV/AIDS in school?**

**Speaker Number three (3):** For me I suggest that the government should bring ARVS in schools and the school nurses should take in charge of them. This will minimize the problem of students to move from schools to pick their tablets in the health centers yet most of them even lack transport to move to such places.

**Speaker Number seven (7):** I was suggesting that those students who are affected by HIV should be identified and counselled about HIV how it is cured, prevented, and treated**.**

**Speaker Number Five (5):** The government should increase ARVS in health centers and bring them in time.

**Qn 10. Is there something you want to add to this interview, something we didn’t talk about but that you feel is important to you and want to share?**

**Speaker Number six (6):** For me I am suggesting that there should be a strong collaboration between health centers in the fight against HIV/AIDS**.**

**Speaker Number four (4):** For me I am suggesting that the government should train doctors and nurses to enforce HIV/AIDS testing in schools to know the number who are living with HIV and start their tablets because if testing is not enforced in schools! HIV will keep on increasing for sure in schools.

**Qn 11. Are there any questions you have for the researcher?**

**Speaker Number eight (8):** Are there types of ARVs?

**Speaker Number seven (7**): I was requesting the government of Uganda most especially the intellectuals, researchers, doctors, and many others look for the medicine of HIV/AIDS so that HIV/AIDS can be cured not just prevented and treated.

**Speaker Number three (3):** Is this research only in secondary schools or even primary schools because there also students with the age bracket that your focused on (15-24) years and they also need a lot of help in regards to HIV care, prevention and treatment services.

## YOUTH IN SCHOOL FGD 3 (YIS FGD3)

**Qun 1**. **Can you describe a typical day in your life?**

**Speaker Number seven (7):** In the lockdown, one day I had my friend we had to go in the outing. Then after going there, we started enjoying our drinks and that friend of mine had a boyfriend and her boyfriend had to tell her that they should go somewhere, and they had the room leaving us enjoying because it was her birthday. After reaching there ‘the room’, they did what they did…. then after two weeks from there, my friend started telling me that I was not sure whether this guy had HIV or not then later., after a month, she told me that she is not feeling okay. Then after we had to say. Do you know what you’re going to do you go at the hospital and test for HIV but besides she feared there [hospital] then we had to give her knowledge of going to the clinic and they tested her they found when, she is HIV positive.

**Speaker Number Four (4**): when it was lockdown, I had my friend and she was HIV positive, she wanted to access ARVs but unfortunately by that time, they had stopped boarder riders from carrying passengers, so she found it very had to go to the hospital to find the ARVS, so she decided to remain in the village.

**Qun2. What do you know about HIV/AIDS?**

**Speaker Number six (6**): HIV is a virus, it can be sexually transmitted through blood and once it enters in the body, it eats up the white blood cells hence weakening the body then it grows into a disease called AIDS and when the time goes on, it weakens the whole body and acquires every disease. Therefore, most of AIDS patients acquires every disease because their body is weak since their white blood cells are also weak. If there is no ARVS to give the body strength, he or she may die due to the weakness and every disease that comes up. Thank you.

**Probe on the commonest mode of spread of HIV mostly in schools**

**Speaker Number one (1**): its mostly through sexual intercourse

**Speaker Number Eight (8):**it can be spread through blood contact especially when using sharp objects with an infected person

**Qun3. What challenges do youth living with HIV/AIDS face?**

**Speaker number two (2):** An example I can give, my friend whom I studied with in school most times kale he was discriminated by her fellow students and also was afraid for it mentally because she would feel as if she were not to be with others.

**Speaker Number Eight (8):** if you find that like one has AIDS in school, he or she feel shame to go in the staff quarters to ask for to get the medicine [ARVs] so this affects the student to get the treatment.

**Speaker Number six (6):** They be afraid to take the medicine in time especially when it reaches time and sees that there are other students around so he/she fears to be looked at and so he/she decide to keep the and waits all of them to dismiss and then she comes back to take the medicine and by that period, time would have gone. Therefore, the patient takes the medicine late for the fear to be laughed at by other students.

**Probe on the poverty as a challenge to the youth living with HIV at school**

**Speaker number four (4):** poverty can be a challenge to the youth living with AIV because you may find that he/she wants to access the ARVS, and the hospital is not in the school and then you find that he /she doesn’t have money to transport him/her to the nearby health center because of poverty**.**

**Speaker Number Seven (7**):it can be difficult to the student living with HIV to buy balanced diet which will lead him or her to weaken his or her body more because of poverty.

**Qun 4. What solutions would you suggest tackling the above challenges?**

**Speaker Number six(6):**we should advice youth that are not living with HIV not to discriminate the youth Living with HIV/AIDS at school and the patients not to fear other students who are not affected by HIV because its Normal tough… it is not particularly normal, a student who is HIV positive may look healthy outside ,it may help so if you see someone who is HIV positive trying to hide himself/herself when taking ARVS, just advise him /her and in case you find that she /he lucks something and you can help, just surrender it to him or her to support the victim.

**Speaker number two(2):**what could be the solution is that we fellow students, we should befriend and not a sure them that being with that disease is not a curse lets love them like the way how we love each other and another thing the school admin should always give care to HIV patients by giving them permission to go and pick the ARVS outside and also to give them a place where to keep their medicine so that they can take the medicine aside from other fellow students who may see it as not good to them.

**Qun 5. In what ways do you think you can protect yourself from getting HIV?**

**Speaker number (7):** As we youth, we should abstain from sex because that is the first method that easily spread sex**.**

**Speaker Number one (1):** since some youth cannot abstain completely from sex, because they are naturally a dictated to that intercourse, they can use other methods like use of condoms under the guideline of doctors and others on how to practice it very well.

**Speaker Number four (4):** Another way of preventing from HIV is by avoiding sharing sharp instruments like razor blade.

**Speaker Number Six (6):** Regular checkups because if you find that he/she is on ARVs, they say that the virus is weak and if you find that he/she is not on ARVS, don’t go with him/her for sexual intercourse.

**Speaker Number five (5):** For me I suggest that there should be some organizations in schools and clubs to educate students on how HIV can be cured, treated, and prevented so that those who don’t know also knows and also counsel those who have it, to live in good terms socially with others so that they don’t feel ashamed. yeah... Those educative programs should be brought near to the students.

**Probe on structural measures to prevent HIV such as laws for students living with HIV to be protected in schools**

**Speaker number seven (7):** yeah…. It’s there but it generalizes both students. Its states that when caught discriminating other students, you get a punishment or sometimes if its serious, your suspended for two weeks and come back with a parent.

**Qun 6. In what ways do you think youth in school get knowledge on HIV prevention strategies?**

**Speaker Number four (4):** Here at this school, we are always exposed to these peachy messages like abstain from sex is there**.**

**Speaker number seven (7):** As we students here, our senior men and senior women, they normally gather us and tell us about the methods on how to prevent ourselves from HIV/AIDS.

**Qun 7. Tell me in which situations one may be prompted to test for HIV**

**Speaker number one (1)**: youth normally go to test for HIV when they are really forced for the fear that they may find them infected with HIV/AIDS. So, when you have someone that you need him /her to test, you have to force her, or you can even escort him /her to the health facility in order to test for HIV/AIDS.

**Speaker Number five (5)**: Another situation that prompts the youth to test for HIV is when he /she feels that the health status is weak every time, then you ask him/her that what’s wrong with you then he/she decides to go for medical checkup.

**Speaker Number six (6):** Another way is group influence, if you have friends and one of them says, do you know what, lets go and test for HIV/AIDS and most of them, the biggest number agrees in the group. Those who don’t want are also forced to go with them and if you don’t want, they decide you to eliminate in the group so he or she is forced to go with them for medical checkups.

**Probe on the effect of stigma on HIV patients at school**

**Speaker Number two (2):** The most effect which those students get is that they don’t have friends and some when they get the disease and their parents don’t know it, they are accused badly by their parents, and some are being left away by their parents for most especially those students who get a disease when their family members don’t have it. The parents get annoyed of the child and end up leaving the child in a very bad-mannered state.

**Speaker Number seven(7)**: Those patients when they get a problem[HIV/AIDS], they don’t get enough care ,**how?** this is when you get your disease from where and your family members don’t know it and it becomes hard to tell your family members that your affected by HIV which means that you will not be getting enough care and that person will not be helped any which means that he/she will be suffering alone and he will not get money to buy good food because your at home…,they cook one meal…which will not benefit your health and no changing because you don’t have money.

**Speaker Number four (4**): You find that someone with AIDS because of being accused or blamed, you find that he/she commits like suicide and because of being despised by others and end up committing suicide.

**Speaker Number six (6**): The challenge most of this youth face is that of neglection, that is someone neglects herself/himself. You find that someone has been like a role model. ehh! kale everyone is aspired to be like him /her, and you find that he/she has got the virus. He /she will hate him/herself and those who will be passing by will continue saying that ...ehhh! I wanted to be like that person so he/she hates himself and when he hates himself, will not perform well because he/she will be saying that after all I am going to die.

**Probe on what can be done to those students [HIV positive] who are affected by those challenges**

**Speaker Number six(6**):if you find someone is in a very deep soul or disappointment, of such a virus or disease, you try to advice, help ,convince and when there is a need like to settle the quarrels at home basing on his/her status, you advice the parents and explain to them that even tough someone has acquired the virus, he will not transmit unless is due to blood because most of them think that someone who is suffering from AIDS. I don’t want to use his/her cup, plate, or fork and which is not okay because it will not affect you since it is not blood contact or sexual intercourse. And if there are no such quarrels, discrimination, will help him/her settle with the friends and parents so that the patient feels okay.

**Qun 8. Tell me what you know about treatment of HIV/AIDS**

**Speaker Number five (5):** The only treatment service which is known by everyone after someone has gotten the disease is to tell someone to take the ARVS then those people with money, I hear that there is also an injection for months…. that what I know.

**Speaker Number six (6**): There is also a treatment [lowers her voice]..ehh if someone has the virus..ehh your given some liquid like juice in a jerrycan[not sure of its name] .so you usually take it when it is over, you go back for more.

**Probe on that juice**

Are you sure it’s there?

**Speaker Number six (6)**: Yes

**Speaker number seven (7)**: There is also blood transfusion whereby someone goes for blood like every three months goes at the health facility removes the blood and puts in another blood.

**Qun 9. How best do you think we improve access to the above HIV/AIDS care and treatment especially for those youth living with HIV/AIDS in school?**

**Speaker Number one (1):** Visits in schools can help HIV patients because a student might fear to go to the hospital but what if someone comes and checks them, he can get some confidence to go and get the medicine**.**

**Speaker number six (6):** Most schools don’t provide the treatment like juice made, injection and many others. The schools should support somewhere somehow.

And also, ARVs should be included in the school clinics for those patients who cannot access to go out for the medicine outside the school.

**Speaker Number seven (7)**: The school should put someone who is responsible about students who are HIV positive. How? this is where that person is aware of students who are HIV positive to check who is need for another dose, or that one needs something to improve on their health.

**Qun10. Is there something you want to add to this interview, something we didn’t talk about but that you feel is important to you and want to share?**

**Speaker Number six (6):** yeah…this goes to you as researchers where you find those people in the deep villages who cannot afford the access to these services and don’t know even what is needed to be healthy and needs to be alive. You find that it harder to these people instead they either commits suicide or do anything bad to harm their lives.

So, you as the researchers…ehh I would like to know if you have reached those areas far away to help those such people.

**Speaker Number seven(7):**As you have asked that what can be the solution to solve the above challenges[HIV/AIDS care, treatment and prevention utilization services] me as a student, I see the ministry of heath should help us and send some representatives from the ministry and hospitals to come in schools and share with us knowledge because most of us students we fear to go in the hospitals to face those doctors and nurses because you can see they are older than you so you can fear them.so the ministry of health should help us to send those representatives to check us in our schools .Thank you.

**Qun10. Are there any questions you have for the researcher?**

**Speaker Number five (5):** There is when you find that the mother is affected by AIDS, and she regularly doesn’t take medicine and she is pregnant so what are the possible effects to that unborn baby. Will it be HIV positive or Negative?

**Speaker Number three (3):** Is your research wants to remain in the writings or there is also another strategy of spreading the gospel to the community especially on radios and televisions because some people you many make research and just writes them and not everyone will go in the book shop and buys the book and even if he /she has the book, some don’t even know how to read them?

## YOUTH OUT OF SCHOOL FGD1 (YOS FGD1)

**Qn 1. Can you describe a typical day in your life?**

**Answers ‘**

**Speaker Number two (2):** I remember one day during COVID19, when my friend who was living with HIV had no money for transport to go to the health centre to pick his tablets and also there was a lot of restrictions during that period where all people were restricted from moving from one place to another for the fear of spreading COVID 19 which disrupted many people from getting their ARVS in time.

**Speaker Number four (4**): During that time of COVID19, many people failed to take their tablets in time because they had no way of how they could pick them from their recommended health centres which disrupted many people who were living with HIV during that period.

**Qn 2. What do you know about HIV/AIDS?**

**Speaker Number seven (7):** I know HIV/AIDS is a killer disease**.**

**Speaker number eight (8**): For me I know HIV can’t be cured but if you continue taking your tablets, you can live longer.

**Probe on the commonest mode of HIV in their community**

**Speaker Number three (3**): Through having sex with an infected person.

**Speaker Number two (2):** Through sharing sharp objects with an infected person

**Qn 3**. **What challenges do youth living with HIV/AIDS face?**

**Speaker Number eight (8):** They face a lot of discrimination from other people who are not infected with HIV which is not good.

**Speaker Number Five (5**): They also face a lot of stigma and discrimination from the public.

**Speaker Number two (2**): They also fear to disclose their HIV status to any one which results into fearing to go and get ARVS from the health centers.

**Probe on poverty on how it is a challenge to the people infected with HIV**

**Speaker Number two (2**): Majority of the patients lack transport to take them to the health centers to pick their tablets because of poverty.

**Probe on the nearest health centre and how they adhere to it.**

**Speaker Number five (5):** The health centre is nearer to us that is Lugazi Health Centre four but surprisingly, most of the patients registered in very far distant health centers to pick their tablets for the fear of being laughed at by their neighbours when picking their tablets in Lugazi which would be nearer to them…. everyone in the discussion supported this point.

**Qun4. What solutions would you suggest tackling the above challenges?**

**Speaker Number two (2):** The government should pay councillors to carryout house to house counselling on how to prevent themselves from HIV/AIDS.

The community should stop the habit of discriminating people who are living with HIV instead they should make them their friends in order to feel comfortable in the community.

**Qn 5. In what ways do you think you can protect yourself from getting HIV?**

**Speaker Number four (4):** we should always use condoms when having sex with our loved ones.

**Speaker Number Eight (8):** You should always test for HIV/AIDS before having sex with your loved ones.

**Speaker Number Two (2):** Youth living with HIV should always take their tablets in time and we should also stop the habit of sharing sharp objects.

**Probed on the structural measures like laws protecting an HIV patient from being discriminated**

- All kept silent.

**Qun6. In what ways do you think youth out of school get knowledge on HIV prevention strategies?**

**Speaker Number two (2):** we normally get knowledge on HIV prevention through our VHTs and chairmen LCI in our area…*Nahabwe Monica youth of Kibingo Village lamented.*

**Speaker number three (3):** we normally listen to radios and take advice from our elderly seriously on HIV prevention.

**Speaker Number one (1**): You need to always carryout such community visits and educate us on how we should prevent ourselves from HIV/AIDS.

**Qun7. Tell me in which situations one may be prompted to test for HIV**

**Speaker Number four (4):** When you have sex with someone whom you don’t know his HIV status.

**Speaker Number two (2)**: We as boys, there is when we use one girl in the group without knowing who could be infected with HIV/AIDS. Therefore, at that movement after the action, you have to go and test for HIV/AIDS to know your status.

**Qun8. What do you think is the next step after one has tested positive for HIV?**

**Speaker Number three (3):** If someone tests HIV positive, he /she should go to the nearby health facility and start ARVS immediately**.**

**Speaker Number two (2):** You should get counseling services from Health workers.

**Qn 9. Tell me what you know about treatment of HIV/AIDS.**

**Speaker Number one (1):** I know taking ARVS only as the only treatment of AIDS

**Qun10. What do you think stops some youth from getting treatment for HIV/AIDS?**

**Speaker Number two (2):** Majority of the use fear to take treatment because they don’t want their parents to know that they are infected with HIV yet they were born without it.

**Speaker Number four (4**): Majority of the youth fear to be seen by their fellows while picking ARVS. That’s why most of them move with their bags at the health facility when it’s their time of picking their tablets.

**Qn11. What would you suggest is the best way to overcome these challenges?**

**Speaker Number two (2):** people with HIV should know themselves and share knowledge on how to live with HIV**.**

Parents should not harass their children especially those who get HIV when they are already old by themselves. Instead, they should bring them on board and take care of them when taking their tablets.

**Qn12. How best do you think we improve access to the above HIV/AIDS care and treatment especially for those youth living with HIV/AIDS in out of school?**

**Speaker Number Two (2):** The government should bring ARVS to the VHTS so that they can always distribute them to the youth in the community who are infected with HIV.

The government should Issue a policy whereby people living with HIV/AIDS pick their tablets in their area health centers and avail enough medicine to those health centers.

Health centers should also organise a schedule for the youth only when they should be picking their tablets at the health facility.

**Qn 13. Is there something you want to add to this interview, something we didn’t talk about but that you feel is important to you and want to share?**

**Speaker number four (4**): The government should make research in communities to know the number of people living with HIV in every area and make a follow up on them to see if all of them are getting their tablets in time.

**Qun12. Are there any questions you have for the researcher?**

- All kept silent.

## YOUTH OUT OF SCHOOL FGD2 (YOS FGD2)

**Qun1. Can you describe a typical day in your life?**

- All kept silent

**Qn 2. What do you know about HIV/AIDS?**

**Speaker number eight (8):** For me I think HIV/AIDS is spread through having sex with an infected person.

**Speaker Number five (5):** For me I think HIV/AIDS is spread through sharing sharp objects with an infected person.

**Speaker number seven (7**): For me I think if you drink on the same cup with an infected person, you also get AIV/AIDS.

**Speaker Number two (2):** For me I think when you’re fighting with an infected person and he /she bits you, you can also get infected.

**Probe on the commonest mode of how HIV is spread in their community**

**Speaker Number five (5):** Praying un protected sex with an infected person is the most common mode of spread of HIV/AIDS in our area.

**Qn3. What challenges do youth living with HIV/AIDS face?**

**Speaker Number eight (8):** For me I think the most challenge they face is lack of medical care from their families.

**Speaker Number seven (7):** For me I think another challenge they face is failure to take their tablets for HIV/AIDS due to stigma.

**Probe on how poverty is a challenge on HIV patients**

**Speaker Number Five (5):** Most of the youth especially girls play sex in order to get money due to poverty end up getting HIV/AIDS.

**Speaker Number seven (7**): Most of the youth lack basic needs from their families which forces them to play sex and end up acquiring HIV/AIDS due to poverty.

**Qun 4. In what ways do you think you can protect yourself from getting HIV?**

**Speaker Number five (5):** You can use a condom while playing sex.

**Speaker Number seven (7):** Abstain from sex**.**

**Speaker number eight (8):** if you have your boyfriend and you want to play sex, you should first test for HIV to know your status before sex.

**Speaker Number two (2):** when you use a condom, you can’t be infected with HIV/AIDS

**Speaker Number one (1):** You need to know the HIV status of your patter before playing sex with him/her.

**Probe on what should be done to prevent HIV**

**Speaker Number two (2**): Abstain from sex

**Speaker Number eight (8**): Test for HIV/AIDS to know your status before sex.

**Qun 5. In what ways do you think youth out of school get knowledge on HIV prevention strategies?**

**Speaker Number seven (7):** we use radios**.**

**Speaker Number eight (8):** From our VHTs.

**Qun6. Tell me in which situations one may be prompted to test for HIV**

**Speaker Number seven (7):** when I play sex with someone whom I don’t trust in regards to his/her status on HIV/AIDS.

**Speaker Number five (5):** For me I think when you play sex with many people whom you don’t know their status in regards to HIV/AIDS Status.

**Speaker Number two (2):** You may not be engaged in playing sex but you my think that you were born with it and this forces you to go and test for HIV/AIDS.

**Qun 7. Tell me why you think some youths do not test for HIV?**

**Speaker number five (5):** Most of the youth fear to test for HIV/AIDS thinking that if they are found with it, they are going to start taking tablets yet they fear the tablets for HIV/AIDS.

**Speaker Number eight (8):** Most of the youth fear to make lines in the hospitals with old people since there are no youth friendly corners which interferes their ability to test for HIV/AIDS.

**Speaker Number seven (7**): Some of the youth fear to test for the HIV/AIDS because most of them think that if they find them infected, they will be chased from their homes by their parents.

**Qun8.What would you suggest is the best way to overcome these challenges?**

**Speaker Number eight (8):** You should always come in our communities and carry sex education so that we also get knowledge on how we can prevent our selves form HIV/AIDS.

**Qun 9. How best do you think we improve access to the above HIV/AIDS care and treatment especially for those youth living with HIV/AIDS out of school?**

**Speaker Number five (5):** For me I think you should also carryout community visits like the way you have come and use VHTS to mobilize the Youth in order to provide us with the information on how to prevent HIV/AIDS.

**Qun 10. Is there something you want to add to this interview, something we didn’t talk about but that you feel is important to you and want to share?**

- All kept silent.

**Qun 11. Are there any questions you have for the researcher?**

**Speaker Number five (5):** Yes, if someone is HIV/AIDS positive and he rapes you and unfortunately you don’t know how to prevent, care, or treat HIV /AIDS yourself, what can you do on that situation?

**Speaker Number eight (8)**: As non-school going youth, what knowledge do you give us so that we can also prevent ourselves from getting HIV/AIDS.

## YOUTH OUT OF SCHOOL FGD3 (YOS FGD3)

**Qn1. Can you describe a typical day in your life?**

**Speaker Number Eight (8**): it was one day when our mother forced us to go to Lugazi health centre to test for HIV/AIDS and she assured us that when they find someone with it, she will make a last party to him/her and chase away from her home because she gave birth children without HIV/AIDS in her home. Everyone feared and we went with her to the health centre.

After testing us, one of our brothers was found with it and the health workers went with him and my mother in a separate room for counselling. This was the day I will never forget in my life.

**Qn 2. What do you know about HIV/AIDS?**

**Speaker Number one (1):** HIV/AIDS can easily kill you especially if you’re not taking ARVS.

**Speaker Number three (3):** For me I know HIV as disease that can make someone lose a lot energy and power.

**Speaker Number Five (5)**: For me I know HIV/AIDS as a disease that can be easily spread through having sexual intercourse and sharing sharp objects with an infected person.

**Qn 3. What challenges do youth living with HIV/AIDS face?**

**Speaker Number eight (8):** Most of the youth face a challenge of stigma. They can’t tell anyone that they are HIV positive and they like spreading it to everyone they come across. They also don’t want to take their tablets.

**Probe on how poverty can be a challenge to the people with HIV/AIDS**

**Speaker number seven (7):** Most of the youth living with HIV/AIDS don’t get basic needs like good food which they are supposed to be taking because of poverty.

**Qn 4. What solutions would you suggest tackling the above challenges?**

**Speaker Number five (5):** we should help those living with HIV/AIDS by giving them financial support so that they can buy what they like.

**Speaker Number six (6):** we should also always make them remember to always take their tablets and counsel them that having HIV is not their end of life.

**Qn5. In what ways do you think you can protect yourself from getting HIV?**

**Speaker number Five (5):** You can abstain from sex**.**

**Speaker number one (1):** I think we should prevent ourselves from sharing sharp objects with people whom we don’t know their HIV/AIDS status.

**Speaker Number seven (7):** If you know that someone has HIV/AIDS, you should prevent yourself from sharing sharp objects with him/her.

**Speaker Number eight (8):** Old people especially grandmothers should stop the traditional way of giving medicine to the young ones by using their mouths because sometimes you find that they are transmitting HIV to the young ones unknowingly especially when they have wounds on their mouth and young ones as well.

**Speaker Number two (2):** Before having sex with your loved ones, you should first test for HIV/AIDS and use condoms as well to prevent your selves from HIV/AIDS.

**Probe on the counseling services in their community**

**Speaker Number two (2**): we don’t have any counseling services in our villages. Though if you find them at the nearby health centers, health workers can council you.

**Probe on the structural measures to prevent HIV like laws that protect people with HIV**

- All were not sure about it.

**Qn6. In what ways do you think youth out of school get knowledge on HIV prevention strategies?**

**Speaker Number six (6):** Some of the youth get knowledge about HIV prevention from radios and social media channels like WhatsApp and Facebook.

**Qun7. Tell me in which situations one may be prompted to test for HIV?**

**Speaker number Three (3):** After having sex with multiple partners whom he/she doesn’t know their HIV status.

**Speaker Number seven (7)**: After having sex with someone whom he/she doesn’t know his or her status.

**Speaker Number five (5):** when you feel that you’re not feeling well in the body.

**Qn 8. Tell me why you think some youths do not test for HIV**.

**Speaker Number Five (**5): They fear that they may find having HIV/AIDS.

**Speaker number seven (**7): Some youth say that if they find having HIV , they will kill themselves instead of start taking medicine.

**Speaker Number two (2):** Some youth wait to see others going to test for HIV/AIDS so that they can also go there. Therefore, if their fellows don’t go for HIV testing, they can’t go there as well.

**Qun8.What do you think is the next step after one has tested positive for HIV?**

**Speaker Number one (7**): For me I think he/she should start on tablets immediately.

**Qn9.Tell me what you know about treatment of HIV/AIDS**

**Speaker number four (4**): Taking ARVS daily.

**Qn 10. How best do you think we improve access to the above HIV/AIDS care and treatment especially for those youth living with HIV/AIDS out of school?**

**Speaker number one (1**): You should always carryout community visits to educate the youth on how they can prevent themselves from HIV/AIDS.

**Qn 11.** **Is there something you want to add to this interview, something we didn’t talk about but that you feel is important to you and want to share?**

**Speaker Number six (6):** There should be mass education to the youth outside the school on how they should test themselves and also avail testing kits at the village level. Even the prices of testing in the health centers should be reduced so that majority of the youth can test at a low cost in the villages.

Even girls don’t want boys to use condoms when having sex for the fear that they don’t get sexual pleasure, that the boy may not put it on well and enters deep in their vagina and cause vaginal cancer. All the above prejudices can’t let them to use condoms which results into easy spread of HIV/AIDS among the youth.

**Qn 12. Are there any questions you have for the researcher?**

**Speaker Number six (6):** How long can HIV strips get expired?

**Speaker Number seven (7**): Is it true that when someone with HIV uses a razor blade and cuts him/her self and last for a long time like 1 hr. That when someone without HIV uses the same razor blade and cuts him/her self, can’t get HIV/AIDS?

# KEY INFORMANT INTERVIEWS

## KEY INFORMANT INTERVIEW1_DISTRCT HEALTH OFFICER (KI11 DHO)

**Qn.** Can you tell me about yourself, about who you are and what you do?

**Interviewee:** I am called XXXXX I work as DHO xxxx local government.

**Qun. What is your opinion on the burden of HIV among the youth (15-24 years) in this community?**

**Interviewee:** it is average, it is not very low and it not very high.

**Qun. What challenges do youth living with HIV/AIDS face?**

**Interviewee:** one is stigma, adherence to drugs is very low, the viral rode suppression is very low. Those are the challenges within that age group.

The above challenges are caused by stigma, and at times they are at school and when it comes to taking drugs, sometimes they forget.

Then their supply for drugs may be irregular because this group at school their drugs may be over, and they will not ask for permission to go and pick drugs because they don’t want to be known.

So those are the things that cause nonadherence to drugs.

**Qun. What solutions would you suggest tackling the above challenges?**

**Interviewee:** The best way to tackle the above challenges is to help them understand that they have a problem and after understanding ,the next step is to accept that they have a problem and then negotiate with them for example if the student is at school and doesn’t have time to pick the medicine, so that we can negotiate to have a supply for the drugs that will last for that period when they are at school.so that negotiation can be possible if they understand and accept that they have a problem. And also set appropriate time when to take that drug so that you’re not seen by anybody. This is the only way how we can solve the problem of nonadherence and low viral suppression.

**Qun. How best do you think we improve access to the above HIV/AIDS care and treatment especially for the youth living with HIV/AIDS out of school and schools?**

**Interviewee:** For the youth out of school, we have already strategies that every health center with in XXXX, receives drugs for these people mostly to that age group.

And more so, we have put programs where they can access the facilities from other facility users for example the occurred hours like 4 pm when others have done his/her businesses to go there as a visitor of course you have to have made communication with the health workers and go there to pick your drugs in secrecy and no one will know it. So, accessibility in Rubirizi is available if someone accepts his/her status.

And the intervention we have put to those in schools is similar to that one for those out of schools and when they have negotiated, they also used the same method to replenish their stock. So, with these strategies, they are able to improve the access of HIV, AIDS care, and treatment services to YLHIV.

**Qun. Is there something you want to add to this interview, something we didn’t talk about but that you feel is important to you and want to share**?

**Interviewee**: yeah ,something that was not talked about is that if your HIV positive, how do you conduct yourself that you don’t get more and you don’t give the others and this is commonly you know HIV goes with sex, so are you able to apply safer sex ,will you will be able to abstain until you get the right partner to marry you need to have mechanisms of preventing from acquiring though you already acquired, but you can reacquire another strain of HIV. And at the end of it all, you become a donor of HIV or a recipient of it.

So, the youth 15-24 is an active age group you may need to have mechanisms of safer sex during their time and negotiate very well with them. There is not this business of getting swing into the direction of finding yourself gone into your life and having more precaution in your life. So that one should be put there as another information that you need to explore from different respondents.

**Qun. Are there any questions you have for the researcher?**

**Interviewee:** Not really.

## KEY INFORMANT INTERVIEW2_DISTRICT COMMUNITY DEVELOPMENT OFFICER (KII2 DCDO)

**DATE:16/03/2022**

**Qun.** **Can you tell me about yourself, about who you are and what you do at this District?**

**Interviewee:** My name is XXXXX, I am a senior District community Development officer in charge of Gender and People with Disabilities in the District**.**

**Qun. How do you know the Burden of HIV among the youth in your community?**

**Interviewee:** When you talk about the burden of HIV in this community, I categories them as follows; Youth headed families and these are the youth who lost their parents, we have those youth who are out of school because they are sick, we have the vulnerable youth basically those who don’t have any care. So, when we talk about the burden, we look at all of those and we still have a high percentage of adolescents when we go to the national standards because basing on the ministry of health statistics it is 12% which is still a challenge. And when you come to our district xxxx, we are the second district with the highest percentage of youth living with HIV apart from Kalangala and Kampala city but when you go to the Rural districts are bad because we have many fish landing sites.

And the highest burden is on youth out of school because they live in high-risk areas, they are bar attendants, others grown in fishing villages.so the out of school youth are at the highest risk.

The most mode of spread is sexual intercourse because they have sex for survival as I told you they are bar attendants, they are hotel girls, so they do sex because they want to survive.

And poverty is the leading factor of all those burdens because when you look at a young child at around 13-14 years droops out of school, the parents don’t have enough care and of course if she is a girl child, she has to go and work as a house maid and the employer will demand sex ,if the wife is not at home, and if she is working in the bar she will be given beer and become intoxicated, and therefore she will enter into risky sex. So, poverty is the leading cause of HIV.

**Qun. What solutions would you suggest tackling the above challenges?**

**Interviewee:** Economic empowerment if we can start some good programs that focus on youth like skilling. Yes, the youth could have been left school at an early age, but he/she have hands, she can go for saloon, carpentry, welding, and many others. So, they can be supported so that they can get something to survive on other than depending on support from the non-related parents.

The government should also reduce on the cost of education though we have UPE and USE, but the cost is still very high in rural communities. So, we still need approaches that can favor the rural communities.

**Qun. In what ways do you think youth out of school and in schools get knowledge on HIV prevention strategies?**

**Interviewee:** In school, they have some associations, they have the classroom teaching according to the curriculum, and also have counsellors and if it is church founded school, there is sometimes when the priest comes.

We also go there because we have community development services in those schools then we also have health assistants, whose role is checking drugs. Thats how they access services in schools.

Those out of school, they get information from Bibanda because before they start watching football matches, they first have some information on how to prevent HIV/AIDS and others they get some information from social gathering like churches.

**Qun. How do most of these youth in school get to know about their HIV status**

**Interviewee:** we normally organize testing services in schools, and we believe they do test and for those out of school, they are already engaged in production and when they are pregnant, they have to automatically go at the health center for testing and sometimes forced to go with their partners. So at least there is testing for youth out of schools.

**Probe as a DCDO if he has ever gathered the youth in the communities to give them information about HIV care, prevention, and treatment services**

**Interviewee:** we always call them for general meetings if we want to pass information for productivity, and other services and then from there, we continue and give the information about HIV care, prevention, and treatment services because AIDS is cross cutting.

And currently, we are using community sports because youth like sports. So, when we organize those events, we must tell them this information about their health.

**Probe on if Youth out of school living with HIV access easily services**

**Interviewee**: Not at all because of two factors; one service and age go together because they also want their peer to serve them.so we are saying that it will be good if all health centers have a peer system especially office for the youth so that they know where they can meet their fellow youth and get services because it is still a general issue so there is still limited access of those youth out of school and even in the schools they still have this problem though they have matrons and senior men in charge of them, they are not yet properly trained well to handle them because they even stigmatize them more that is they normally go on the assembly and say ‘the rest go and we remain with those with HIV ‘such statements even stigmatizes more YLHIV in schools. But I am told that you have a program here called HAY which is helping us at least.

And on the health facilities, we have services for them but the process of accessing services for the youth still have bottlenecks because of the above challenges.

**Qun. How best do you think we improve access to the above HIV/AIDS care and treatment especially for the YLHIV in schools and out of schools?**

**Interviewee:** peer service access so that the youth recruit’s youth and train them to help their youth. we should also give special attention to the youth at the health centers by designing a special allocation for them and attended there because there is a way how youth react. They can’t wait for longer hours.so to avoid such embarrassment, there should be special attention for them**.**

We should have community workers for the youth at the village level not the VHTS. we should have mobile youth health workers for the youth train them and give them what is necessarily like vehicles, Laptops, testing kits, CDS and move in the community to give services to the fellow youths in a hamble way.

We should also empower households to look after their youth who are living with HIV in terms of giving them good food and other services needed by them in order to improve on the adherence for the drugs. So, we should empower the youth in order to get what to do for themselves.

**Qun. Is there something you want to add to this interview, something we didn’t talk about but that you feel is important to you and want to share?**

**Interviewee:** we should change the mode of operation because we are still penalizing HIV, this is a disease like others, we don’t penalize malaria, yet it is also a disease.

At this time, we see someone with HIV like a criminal and let me tell you, you cannot fight a disease using criminal approach because it means if you use a criminal approach, the infected ones will hide themselves and, in the hideouts, they will be transmitting it. So, we should reduce on the criminalization of HIV/AIDS.

If there is new technology, we should reduce in the management of drugs because it is not easy for a person to take the drug every day. They should at least make a drug that lasts for two weeks or a month and many others because we have to socialize.

So, if we can improve on the technology for someone to widen the gap of takin the medicine so that it becomes easy and free.

And finally, if you can get donors to support us in supplying the drugs and also economic activities so that when we get a YLHIV we supply him/her with like two rabbits to improve on her welfare.

**Qun. Are there any questions you have for the researcher?**

**Interviewee:** You have taken my voice; will I hear my voice again?

## KEY INFORMANT INTERVIEW3_ DISTRICT EDUCATION OFFICER (KII3)

**Qun. Can you tell me about yourself, about who you are and what you do at this school?**

**Interviewee:** My name is XXXXX. I am the District Education Officer (DEO) for district xxxx local government.

**Qun.AS a DEO, what is your opinion on the burden of HIV among the youth (15-24 years) in this community?**

**Interviewee**: it is huge! Because the rate at which HIV is spreading is very high. There are many issues which we need to take in the considerations’, one there is a problem of broken families bringing what we call child parents and child parents of course have no control over the children under the child parents. The child parents are brought by broken families due to lose of parents because of HIV.

So, from 1985, to the present day, the situation has been increasing on the families without heads they either the father died, or the mother is sick, or both died, and they find themselves not having anyone to take over the family. And this informs us on the burden we are having on the children droop out because they don’t have what to support themselves at school like food, clothing’s, medical care, and scholastic materials. The medical care has been isolated because there is now some care a bout ARVS but for people to come out and start taking ARVS, is another problem because you find people in Rubirizi getting from Kyamuhunga, Bwera which is very far.

And now you find that child born of HIV even fear to take her child on the treatment because she doesn’t want to be known that she was testing her child and is going to take the ARVS. There is a big problem and so when you talk of the burden, there is a big problem.

So, you and me and other stakeholders needs to be aware but aware and awareness is in the seek of ‘awareness’. Therefore, internalising awareness is also another problem. Yes, people are aware, but they have not internalised the awareness to make positive change towards in behaviour, reaction towards what may be given by the government and other NGOS who can assist so that they come and join those children who may have been affected without their consent.

Another this is that drug abuse can also be attributed to stigmatization and lose of hope from the YLHIV.so the level of awareness should be clear to them by taking information the way it is.

**Qun. What solutions would you suggest tackling the above challenges?**

**Interviewee:** The government should take a strong stand against drug abuse of any kind like alcohol, marijuana, and many other intoxicating drinks of higher level.so we need a new study of drug abuse of any form.

The families which still have heads should take kin looking at the children because technology has increased the problem that is phonography which is now uncontrolled according to my thinking.

The other one is to sort out the issue of child up breeding .it not a one day to sort out that, we should start on child rearing practices and child up breeding .so the state should invest more than that is doing in primary education.

The state should enforce parents to do the roles they are supposed to do in the Education act which states that:

The state should be doing the following

- Provision of education and training to a child.
- Provision of learning and institutional materials and many others.

The parents should be doing the following

- The parents have not done what they are supposed to do in the education sector like food, moral up breeding, clothes, shelter which exposes their children at a high risk of getting HIV/AIDS.

**Qun. How best do you think we improve access to the above HIV/AIDS care and treatment especially for the YLHIV in school?**

**Interviewee:** if we properly handle the role of parents and have a provision of those children without parents to reduce the permissiveness of parents saying that the government is looking after our children. And for those youth whose parents are not alive should be properly well handled which we are lucking. So, I think is a need for the government to limit the number of children each family should produce because much as producing a child is a human right, but it is not a human right to produce and neglect a child!.so the government can say that produce the number of children you want but take care of those children produced and therefor the right way for UPE and USE should have been the number of children as it had started for each family to have only four children enrolled. And if you cannot take care of other children, you should be imprisoned because it not your right to bring someone in life and you don’t struggle for him/her.

The government should find means of taking care of those children who do not have their parents. So, if we achieve that, then we can sort out some of the problems.

**Qun. Is there something you want to add to this interview, something we didn’t talk about but that you feel is important to you and want to share?**

**Interviewee:** one thing which is important is to pin down the parent to carry his burden and not just to carry his burden any how he wants. You know politics has disrupted everything because those government leaders keep quite because they want votes but for us technocrats we are saying if you have brought someone to us, let it be legal that you have no right to give the care below human requirement.

The state should make an extra mind to make people contribute towards their children like we should have of an educational tax so that the government takes care of their children.so that government take care of those parents who cannot take care of their children.

There should be not right to have anybody not to be educated so that we don’t have a group that is not in the school.so there should be compulsory free primary Education and Secondary Education because there is a big problem in not having it.

**Qun. Are there any questions you have for the researcher?**

**Interviewee:** since your researchers, we are looking for partners in education.it can be good if we pattern with you?

## KEY INFORMANT INTERVIEW4_DISTRICT FOCAL PERSON IN CHARGE OF HIV/AIDS (KII4)

**Qn. Can you tell me about yourself, about who you are and what you do?**

**Interviewee:** I am called XXXX, I work as an HIV educator, but I coordinate HIV activities for the district.

**Qun. Can you describe a typical day in your life as a District focal person in charge of HIV/AIDS?**

**Interviewee:** I interface with Youth almost on a daily basis because for HIV you can’t tell that this one is positive or negate unless you test him /her, and they normally come here and I interact with them whether I am aware that they are HIV positive or not aware, remember! it is something [HIV] that people keep to themselves since they have stigma because that is their personal issues. So that there is no way how I can say that I don’t meet them, but I probably don’t know that they are HIV positive or negative. And the fact that I am the district focal person in charge of HIV, they normally come her, and I am social to them, and I normally guide them where to go and link them to the health facilities.

**Qun. What is your opinion on the burden of HIV among the youth (15-24 years) in this community?**

**Interviewee:** Actually, it is a big thing 15-24! these are the people we see from the data we have HIV/AIDS. Rember that they are more productive, they are young, and they are still enjoying, and they actually have HIV, but the good thing is that for those ones who are married, because that age group is tricky since some of them are in schools. Others are married, and others not in school. For the, married one, they adhere to the HIV treatment services. But for these ones who are still in schools, they have a lot of stigmas. There is no clear guidance on how these people should take on their drugs.so they still have a lot of stigmas, and they are in schools and taking drugs when other is seeing you is not easy and becomes tricky to them. But if we can organize a proper strategy to handle them, they can adhere to treatment.

But these ones who are not in schools, they have issues for not testing for viral rode, stigma, and many other. They have many issues actually than those one in school.

Generally, the age category is all at the risk according to the data that we have at the district.

**Qun. What solutions would you suggest tackling the above challenges?**

**Interviewee:** continuous health education to curb this stigma because stigma is tricky because there is this external stigma that come from the community and there is self-stigma which comes within the person because even if you talk and talk, the person will think that you’re talking about him/her yet you’re not meaning him/her, and this continues to kill people.

To the school going ones, there should be a system is put in schools to be able to counsel these people and to cater for themselves so that they can take their medicines because when it comes to schools, it becomes very tricky to them since they stigmatize themselves because even these teachers in charge of them, they don’t keep their secrets which stigmatizes them more.so there should be continuous awareness in the community so that they should not stigmatize them and even to the person living with HIV to reduce self-stigma.

We should also inform people that HIV does not kill people very first as long as you take your medicine and adhere to the treatment.

For those people that do not have stigma is a normal disease but for those with stigma, it becomes had to them to stay with it.

**Qun. What do you think is the proportion of the youth that test positive that adhere to treatment?**

**Interviewee:** I think I have also tackled that in this way, I said that for those one that are not in schools and are married, it is easier for them to adhere because they are at home, and they know that they are HIV positive.

But for these ones that are not in schools, it is tricky because they don’t want to take their drugs when their friends are seeing them for example some is at the university and still looking for a boyfriend or girlfriend and you’re saying that he/she opens and takes his /her drugs! It becomes had for him/her to adhere.

**Qun. How best do you think we improve access to the above HIV/AIDS care and treatment especially for the youth living with HIV/AIDS out of school and in schools?**

**Interviewee:** I think for Rubirizi, we have done very well because almost every facility has ARVS and for those who are willing to start treatment, they have access to the treatment however, the gap is still on privacy for those HIV patients at the health facility since not everyone with HIV wants to be seen at the health facility. You find our facilities have small space, we don’t have adolescent and youth corners, it’s now recent that the health workers are finding means of these youth to come at the evening but also depends on the arrangement of a health worker since they are those who may say that come at any time you wish. This is because we don’t have enough space and infrastructure.

There is also a challenge of knowledge gap. Youth do not know other treatment services like PEP and pothers

So, some of these challenges we have are not for health workers, they are for the government. I should say.

We also have a challenge of stock out and sometimes they don’t deliver the drugs in time though we normally go to other districts and to the implementing patterner, but we find this very tricky to us.

**Qun. Is there something you want to add to this interview, something we didn’t talk about but that you feel is important to you and want to share?**

**Interviewee:** I wanted to ask why you are focusing on only 15-24 years because for me I see the biggest challenge of HIV/AIDS spread is even below that age bracket. Children 5-6 years, even they don’t know that they are born with it, will others get it from the maids. So, for them they are more vulnerable and even the parents know it but they don’t even want to take the children because they are stigmatized themselves and they can not adhere to the treatment. So, I think 15-24 years when educated very well, they can man over but these young ones 5-6 years! There is a big problem to them.

**Qun. Are there any questions you have for the researcher?**

**Interviewee:** Will you share with us the research findings?

# IN-DEPTH INTERVIEWS

## INDEPTH INTERVIEW NUMBER1_HEALTH WORKER (IDI_HW01)

**QUN. Can you tell me about yourself, about who you are and what you do?**

**Interviewee:** I am a midwife by profession, I am XXXX working at XXXXX Health center two.

**Qun. Can you describe a typical day in your life as a health worker?**

**Interviewee:** since this one is a health center two and Art has just started, currently we don’t have youth that are accessing art services here and even the children we don’t have we have only the adult.so the clients mostly which I see here are above the age .The youth I have[low voice] are the PMCT mothers of which I take them as adults however, I have not seen any challenge with them only that the main challenge I saw, is the disclosure is the most problem to their patterner and especially to their family members. Nothing much I have seen there because they are complying well with their treatment.

**Qun. What is your opinion on the burden of HIV among the youth (15-24 years) in this community?**

**Interviewee:** what I have seen youth because of fear, shame, they don’t normally to access have services as testing is concerned because they think that a youth should not access the art services when the older are there so HIV testing is still very low among the youth hence making it for them very possible to acquire HIV/AIDS.

Another thing is that stigma remains still a problem and because of this pandemic that have been there, it has put the youth on the high risk of acquiring HIV because there has been redundancy among youth and yet since they fear to be seen at the Health facility, they hide from there, the few who has come, are those who got pregnant or those who got married are the ones which access services and also because of the pandemic that happened, even there is low levels of education among the youth, the is a high risk of acquiring HIV and even the parents who have low levels of educations makes their children at a high risk of acquiring HIV/AIDS.

Those who youth who live in villages are at the highest rate of acquiring HIV those who live in schools are protected but those in the villages, are not. Since they have high chances of playing sex with the old people, youth. Therefore, the youth who are not at school, they are prone to HIV/AIDS.

**Qun. What challenges do youth living with HIV/AIDS face?**

**Interviewee:** The challenges as earlier said, first of all they fear to test and at any one point of time, when they come and test and find when they are HIV positive, chances of adherence to treatment are very low. Most of them, they will disappear along the way, and they will not continue with the treatment since they are not friendly to the health facility and also disclosure is also a problem to them.

And another thing is that most of our health facilities we don’t have youth friendly corners which means that we fail how to give youth services.

**Probe on how poverty is a challenge to the youth Living with HIV/AIDS**

**Interviewee:** Yes, because most of the youth go there to get money and at the end, they end up getting HIV/AIDS.

**Qun. What solutions would you suggest tackling the above challenges?**

**Interviewee:** First of all, we need to create youth create youth friendly corners so that they know that there is a certain day when youth can go at the health facility and access services. Then another thing is that we need to strengthen education because it is very key since if they are educated, they make the right decision.

For those non-school going youth if they can be given income generating activities so that so that this system of going to the adult people yet they may be infected, so if they are given income generating activities, they may not go to seek for money from the older people.

Then another thing I think we can get like peer groups at the community levels so that they can spread the message over themselves so that they can remove fear from them.

We need also to strengthen parent child relationship because some parents do fear their children to tell them these messages concerning HIV/AIDS awareness. The parents saying that ‘pack condoms and go!’, most of the parents do fear.so we need some good messages to pack for the parents so that they can talk to their children as far as HIV/AIDS awareness is concerned.

**Qun. In what ways do you think youth in school get knowledge on HIV prevention strategies?**

Interviewee: They do get information from the teachers. Then at the health facility, we also do HIV prevention services because we have the place where we put condoms and also, we give messages about HIV Prevention to those schools like ABC.

**Qun. In what ways do you think youth out of school get knowledge on HIV prevention strategies?**

**Interviewee:** They have been getting information from the VHTS, and even from radio stations on those programmes that target them. And also, we give messages at the health facility as far as HIV is concerned.

**Qun. How do most of these youth get to know about their HIV status?**

**Interviewee:** I normally see those youth who are out of schools because the time I have spent here, they are now friendly at me. But they don’t normally come when the patients are there[low voice and laughed] when the patients have gone, I normally see them and they say ’Musaho,I want to test for HIV’I have noticed that they want their own time speculated for them for testing.

**Probe on the challenges to testing services on the above group**

**Interviewee:** The only challenge is that they fear to be seen at the health facility and also to find that they are HIV positive because there was one time when the youth asked me that ‘if you find that I am HIV positive, what would you do to me and I said that I will declare the results to you and she said that if you find that I am HIV positive, don’t give me the result slip that is positive. Give the result slip that is HIV negative because my parents are going to ask me’ so they are in that dilemma that when they are tested positive, it will be hard for them.

**Qun. What is the process of enrolling the youth into treatment after testing positive?**

**Interviewee:** Before we test, we do pretest counseling, then we test, then after the results, we do posttest counseling, then from there, we have our registers and we really discus with the youth. We ask him/her that would you wish to be getting the drugs from here, who are you going to disclose too and many others.

**Qn. What do you think is the proportion of the youth that test positive that adhere to treatment?**

**Interviewee:** since I don’t have the youth, but the PMCT mothers I have, have been complying very well to their treatment because they wish not to transmit their HIV to the babies.

**Probe on male involvement with PMCT mothers in the Ant- natal care**

**Interviewee**: Male involvement is still very low.

**Qun. What do you think are the challenges to adherence among the youth?**

**Interviewee:** I don’t have any challenge with those PMCT mothers. The only challenge I have is male involvement testing with them.

**Qun. What would you suggest is the best way to tackle these challenges?**

**Interviewee:** it would be better to first ask permission from these mothers because I would go there, remember these mothers have not disclosed to their husbands which may cause more problems. But it would be good if the mother tells her husband about their status.

**Qun. How best do you think we improve access to the above HIV/AIDS care and treatment especially for the youth living with HIV/AIDS out of school?**

**Interviewee:** we can have outreaches for youth only so that they are youth only there will come. Then another thing is that we should have a youth friendly corner for the youth.

We should also establish youth peer groups and they work with us at the facility and also work in the community because it is easy for a peer to encounter the youth at the village level.

**Qun. What could facilitate better utilization of HIV/AIDS care, treatment, and prevention services for the youth out of school?**

**Interviewee**: All are the same

**Qun. Is there something you want to add to this interview, something we didn’t talk about but that you feel is important to you and want to share?**

**Interviewee:** Not really

**Qun. Are there any questions you have for the researcher?**

**Interviewee:** Yes, after this research, are you going to share the information to the communities or to the NGOs**?**

## INDEPTH INTERVIEW NUMBER TWO_HEALTH WORKER (IDI HW02)

**QUN:** **Can you tell me about yourself, about who you are and what you do**

**Interviewee:** I am a health worker here

**Qun. Can you describe a typical day in your life as a health worker?**

**Interviewee:** For us here HIV services we offer them on Thursday and there is no typical day for me because I know how to counsel by patients**.**

**Qun. What is your opinion on the burden of HIV among the youth (15-24 years) in this community?**

**Interviewee:** Both school going and non-school going youth are highly affected in this area and you can not specify.

And the commonest mode of spread of HIV/AIDS is sexual intercourse**.**

**Qun. What challenges do youth living with HIV/AIDS face?**

**Interviewee:** There are many challenges one is stigma, finance, youth don’t have enough care, there is violence, some are orphans. think those are the most challenges which they face.

**Qun. What solutions would you suggest tackling the above challenges?**

**Interviewee:** one before we get a medicine that can prevent HIV/AIDS, there will be no solutions to these challenges. The challenges will even increase more because finically you cannot give people money, for orphans there should be organizations to help those orphans.

**Qun. In what ways do you think youth in school get knowledge on HIV prevention strategies?**

**Interviewee:** some of them are not aware of these methods, they no condoms but most of them don’t know even how to use them and few have attended those educations teaching them on how to use them. And that is the biggest challenge.

**Qun. In what ways do you think youth out of school get knowledge on HIV prevention strategies?**

**Interviewee:** Even those out of schools are also not aware of these methods and are not even very many according to the age bracket your giving me. unless most of them were born with HIV/AIDS but most of them are in schools.

**Qun. How do most of these youth get to know about their HIV status?**

**Interviewee:** Ideally, we advise the youth to come for HIV/AIDS testing, but it is still hard for these youth to go for testing. Unless if they are exposed like someone has slept with the person, he/she is not sure about his/her HIV status we normally see them. But their seeking behaviour for the youth to come for testing is still very low.

**Probe on the challenge of these youth to HIV/AIDS testing services**

**Interviewee**: I think the most challenge is stigma because one fears to say that how should I say that they should test me.

**Qun. What is the process of enrolling the youth into treatment after testing positive?**

**Interviewee:** Before we test, we do a pre-HIV/AIDS counselling, then we test, then we do a post HIV counseling and if someone has tested positive, we do counsel again, then after we start on treatment**.**

**Qun. What do you think is the proportion of the youth that test positive that adhere to treatment?**

**Interviewee:** Adherence is very well who are taking their drugs but the youth that are testing and testing positive, our rate is still very low and the challenge I think is that most youth do not go for HIV testing.

**Qun. What do you think are the challenges to adherence among the youth?**

**Interviewee:** As I told you, most of these challenges are stigma and financial (poverty)

**Qun.** **What would you suggest is the best way to tackle these challenges?**

**Interviewee:** To tackle the above challenges is very hard because most of the youth get HIV/AIDS because of money. The most ideal to tackle those challenges is prevention because you cannot control them.

**Qun. How best do you think we improve access to the above HIV/AIDS care and treatment especially for the youth living with HIV/AIDS out of school?**

**Interviewee:** The most important thing is creating awareness by making sure these youth get the write information that is through churches, radios, and also encourage them because it looks bad to say that everyone who comes at the health facility we test because sometimes you may find that we use some of our strips that are not meeting the target.

**Qun. Is there something you want to add to this interview, something we didn’t talk about but that you feel is important to you and want to share?**

**Interviewee:** its fine, and I have liked the research that your carrying out but ideally about adolescents, you know from 15-24 years is the target age group that’s were most people get HIV and according to this population, it’s like four times for the younger girls than the younger boys for the same age bracket and when we check at the rate at which HIV is spreading, it is four times for the younger girls than for boys. Now when we are looking here, I don’t know where we should put most of the emphasis. For me I should prefer us to put more emphasis on the younger girls than boys of the above age bracket.

**Qun. Are there any questions you have for the researcher?**

**Interviewee:** I would like to know the end result of this information that your collecting, is it going to help the community? is it going to end here? is it going to come with an NGO that is going to support the youth?

## INDEPTH INTERVIEW THREE_HEALTH WORKERS (IDI HW03)

**Qun. Can you tell me about yourself, about who you are and what you do?**

**Interviewee:** I am XXXXX I work with Rubirizi Local government posted here at XXXX Health Center four and I am in charge of the ART clinic.

**Qun. Can you describe a typical day in your life as a health worker?**

**Interviewee:** it was one day when I had a task of counseling one youth, and this youth was not disclosed up to the age of sixteen years why he was taking the treatment. He was born with HIV, and the mum kept him on treatment. The mum only told the boy that you have some virus in the body and if you take this treatment, this virus will one time heal so you have to keep taking it and if you don’t take, you will die. So, the boy only became used to that word. Only recently when the boy was put in a boarding school, and the drug was brought to the school nurse, so the friend saw the boy everyday going to the school nurse to take the drugs and saw the tin of that drug. Then on reaching the dooms, they asked the boys that are you taking ARVs? And he narrated the story of what her mum told him. So, he came to us to know if it was HIV. After telling him that it was HIV, disclosing to him that it was HIV was a typical day for me.

**Qun. What is your opinion on the burden of HIV among the youth (15-24 years) in this community?**

**Interviewee:** Stigma is the most burden because youth want to feel one another, they don’t want their friends that they are HIV positive, they just want to fit in the group that he/she is not known that he/she is taking the drugs so that their status is not disclosed.

And the most affected group is the non-schooling youth because when you find a youth at home not studying, he/she must have problems like orphanage, poverty, food scarcity and many others which increases the risk for them to have high viral rode.

But for school going, it is different because these youth are Enlighted about HIV, some of them know their status, they always have their treatment.so non-school going are in danger.

**Qun. What challenges do youth living with HIV/AIDS face?**

**Interviewee:** They have family challenges like poverty, team challenge of disclosure, and health care challenge like they want to come at their own time. When other patients are there, they can’t come, and they want to be served very first when they come at the facility. They can’t over wait. They also don’t want to be seen carrying those drugs.

**Probe on how poverty is a challenge to those youth Living with HIV**

**Interviewee:** yeah, because most of them luck transport to come for drugs, and also leads him/her not to get what he/she wants as an individual which becomes the biggest challenge to them.it also leads to poor adherence for the drugs because someone has not eaten.

**Qun. What solutions would you suggest tackling the above challenges?**

**Interviewee:** we need to come up with contact trussing and identification of families that have HIV patients like them to some organizations that can help them.

We should also put restrictions on some drugs that should not be taken to HIV patients. And also serve the youth faster when they come at the health facility such that we can achieve the goal of them adhering to the treatment.

And also, health workers should be active listeners, to these youth because sometimes they have a lot to share with them, but we don’t give them a lot of time.

They also need continuous counseling, continuous support and also avoid discrimination and also tell them encouraging statements.

**Qun. In what ways do you think youth in school and out of schools get knowledge on HIV prevention strategies?**

**Interviewee:** Those out of school, they get knowledge through community visits of our groups, radio talk shows.

And those in schools, they are always some posters in schools that talks about awareness.

Most of them are attached to the health workers which provides information to them.

**Qun. How do most of these youth get to know about their HIV status?**

**Interviewee:** Most school going are the ones who normally test for HIV mostly when we go there for school visits.

**Qun. What is the process of enrolling the youth into treatment after testing positive?**

**Interviewee:** it is the usual process of HIV counseling, and it doesn’t take a lot of time.

**Qun. What do you think is the proportion of the youth that test positive that adhere to treatment**?

**Interviewee**: School going youth because these non-school goers can be enrolled, finish one month and the next months doesn’t come.

**Qun. What do you think are the challenges to adherence among the youth?**

**Interviewee**: stigma and poverty

**Qun. What would you suggest is the best way to tackle these challenges?**

**Interviewee:** We also need to put up open up testing centers for the youth and also enhance HIV self-testing services.

We also need the young peers to counsel not the older ones.

We should also encourage parents to give a chance to HIV youth because they are also people like other if on treatment but not discriminated from home.

**Qun. How best do you think we improve access to the above HIV/AIDS care and treatment especially for the youth living with HIV/AIDS out of school and in schools?**

**Interviewee:** For those out of school is continuous counseling and also encourage family members to be disclosed too so that they can have that child not discriminating him/her from home.

We should also encourage contact tracing and index testing for that family such that for any another positive can be known and put on treatment.

Also, we encourage counseling the family to make sure that the child gets the treatment in time for easy adhering and also, we have community drug distribution points where we have some areas where we need to go inside communities, and we meet them from there.

We can even do home visits if they ask for it or if we see it as a benefit to some of the families.

Then for school going, we can have drug refills whereby we communicate with schools and look for the ways we can send the drugs to them.

**Qun. Is there something you want to add to this interview, something we didn’t talk about but that you feel is important to you and want to share?**

**Interviewee:** This age category needs a lot of support like through NGOs and support groups and encourage health facilities to have special days for this group so that they don’t come with other old people.

We should also encourage facilities to have young peers who are HIV positive to be the ones at the facility to do the counseling and take part in all other services concerning the youth.

We should also emphasis on listening to them and dig deep what this age category is facing so that we can give the best services and good adherence to treatment for them.

**Qun. Are there any questions you have for the researcher?**

**Interviewee:** Is this research going to have support to that age category? is it going to be funded? What will happen to those barriers that you have got out of them?

## INDEPTH INTERVIEW NUMBER ONE_VHT (IDI VHT01)

**QN1. Can you tell me about yourself, about who you are and what you do**?

**Interviewee:** My name is XXXX. I work as a VHT of XXX Village and at the sometime as a VHT coordinator of XXXX Parish.

**Qn 2. Can you describe a typical day in your life as a health worker?**

**Interviewee:** it was one day in the during COVID19 when I meat an HIV infected boy in my area who wanted to commit suicide because of not easily accessing his medicine from his health centre. The boy was accessing his medicine from Kyamuhunga Health centreIII which was far from his home area and by that time it was a lockdown period and nobody was supposed to move. I therefore took him to the nearby heath centre and they issued a document for him to use when he wanted to access his services. This is how I helped the boy. I will never forget that day in my life!

**Probe on how many youths in his village that are suffering from HIV/AIDS**

**Interviewee:** Per now, I know one youth who is on ARVS But I think there are many youths who live with HIV/AIDS in my area because I see majority of them have signs and symptoms of HIV though they fear to go for testing. Otherwise, there are nearby health centers where they can pick their medicine from like Lugazi Health centre four and Twimukye health centre II.

**Probe on the commonest mode of spreading HIV in their area**

**Interviewee:** For me I think it spreads through having sexual intercourse with an infected person and also to have sex with someone whom you don’t know his or her status.

And majority of the youth in towns are sex workers especially girls because they don’t have any alternative of survival in towns.

**Probe on the challenges of youth living with HIV/AIDS in their area**

**Interviewee:** some of the youth commit suicide after testing HIV positive due to fear.

Majority of the youth who are HIV positive fear to take their tablets when others are seeing them which is not good.

Some of the youth who are HIV positive fail to pick their tablets from their health centers due to poverty since most of them even lack transport.

Another challenge is that majority of the patients lack enough education on how they should live peacefully with the Disease for them to live longer.

**Probe on what can be done to tackling the above challenges**

**Interviewee:** You should always carry out community visits to educate our youths on how they should prevent themselves from HIV/AIDS at least every three months.

**Probe on how youth out of school get knowledge about HIV/AIDS**

**Interviewee:** They mostly get such information from radios for example Rubirizi FM.

We as VHTs carryout education programs in our areas on how the youth should prevent themselves from HIV especially when we get a chance in a social gathering like a community meeting, burials and parties.

We as VHTs we normally call councillors from health centers in our communities to carryout HIV testing and education but the problem with this is that majority of the youth don’t want to test for the fear of being infected with HIV.

**Qn 2. What could facilitate better utilization of HIV/AIDS care, treatment, and prevention services for the youth out of school?**

**Interviewee:** I suggest that there should be education programs on how youth can prevent, protect and care for HIV because when COVID19 can, people relaxed on HIV yet it is still there killing people.

**Qn3.Is there something you want to add to this interview, something we didn’t talk about but that you feel is important to you and want to share?**

**Interviewee:** You should always come in our villages and carry out civic education on how the youth should prevent HIV/AIDS and even us VHTs we can carry out role plays and songs regarding to the prevention of HIV/AIDS. we should always have youth meetings specifically on how they can prevent themselves from HIV.

The government should put on more effort on educating the public about HIV prevention because majority of the people have for gotten that even HIV exists which is very dangerous.

## INDEPTH INTERVIEW NUMBER TWO VHT (IDI VHT02)

**Qn1.** **Can you tell me about yourself, about who you are and what you do?**

**Interviewee:** My name is XXXX. I work as a VHT of XXXX parish, XXXX Sub -county, Rubirizi District.

**Qn 2. Can you describe a typical day in your life as a health worker/VHT?**

**Interviewee:** For me what surprised me during this period of COVID 19 is that most of the youth got AIDS and early pregnancy in my area due to a lot of freedom from their families which resorted to redundancy and boredom among them.

Another thing is that majority of the youth fear to test for HIV saying that “how can I tell the doctor that I have come to test for HIV/AIDS” but we as VHTS we try by all means to sensitize them that they should go for HIV testing to know their health status. Therefore, what I disliked during this period of COVID 19 is that majority of the youth didn’t want to test for HIV yet they like to be having fun in risky areas of HIV with sex workers.

**Probe on how many youths in his area that are suffering from HIV/AIDS**

**Interviewee**: per now there is no youth which I know that is HIV positive in my area but I know that they are there though they fear to go for HIV testing. The old people are the ones who are normally go for HIV testing and for them they even adhere to their medicine.

**Probe on the commonest spread of HIV/AIDS in his area**

**Interviewee:** There are many factors that bring out the spread of HIV/AIDS but what is most common in my area is playing sex with an infected person in my area without even protecting yourself and testing him/her to know his /her status.

Another major commonest spread is that majority of the mothers in this area like delivering in their homes which makes them to easily infecting their young ones most especially those who are living with HIV. This is due to fear of high prices charged to them in the health centers after delivering and also long distances from their homes to the nearby health centers.

Other mode of spread is using sharp objects with an infected person and praying sex without using condoms.

**Probe on how poverty is a challenge to the youth living with HIV**

**Interviewee:** Majority of the youth lack basic needs and the only option is to play sex for money especially girls. This results into easy spread of HIV amongst them due to poverty.

Some of the youth lack even transport to go to the health centre to pick their ARVS due to poverty.

**Probe on the challenge of stigma on HIV patients**

**Interviewee:** Most of the youth end up killing themselves due to stigma thinking that people don’t like them which is not the truth.

**Qn. What solutions would you suggest tackling the above challenges?**

**Interviewee:** I suggest that HIV education should be done at the village level because if it remains in the parish, majority of the youth don’t even go there and we should normally have new faces during that education because if they see us educating them, they don’t take it seriously because they are used to us.

We should also counsel those youth who are already positive that they should no be stigmatized because that’s not the end of their lives.

We should also plan very well when we are holding radio talk shows about HIV prevention to target the youths because most of the programs are held when the youths are not available at home or when they are busy doing other work. We should therefore plan to have those radio talk shows especially at 08:00 pm and above when they are already at home.

We should also as VHTs always visit each other when carrying out community education and sensitization because when people see new faces, they normally tell them their secret problems thinking that they will not see them again.

Every VHT in his or her area should get a chance of educating his/her people in the community especially during community meetings and social gathering.

**Qn. How best do you think we improve access to the above HIV/AIDS care and treatment especially for the youth living with HIV/AIDS out of school?**

**Interviewee:** For me I suggest that VHTs should be given transport so that they can go to the health centers and pick the tablets for HIV patients in the area and distribute the medicine to them. This will reduce fear for those youth who fear to pick their medicine from the health center.

**Qn. Is there something you want to add to this interview, something we didn’t talk about but that you feel is important to you and want to share?**

**Interviewee:** I thank you and the organization that has sent you and also request that always come in our communities and educate our youths on how they should prevent themselves from HIV/AIDS because for us as VHTs, they are used to us and can’t take our advice serious.

**Qn. Are there any questions you have for the researcher?**

**Interviewee:** I would like to inquire from you how far with the VHTs allowance that our president told us because we do a lot of work yet we are not paid.

## INDEPTH INTERVIEW NUMBER THREE VHT (IDI VHT03)

**Qun1. Can you tell me about yourself, about who you are and what you do**?

**Interviewee**: My name is XXXX. I am a VHT of XXXX cell, XXXX Sub County in xxxx District.

**Qn 2. Can you describe a typical day in your life as a health worker?**

**Interviewee:** it was one day during COVID 19 when I found a boy playing sex with my neighbour’s daughter but when I told my neighbour that her daughter was playing sex with the boy in the banana plantation, her parent told me that her daughter is old enough and can now make her own decision. This taught me that most of the parents are the ones which make their children indulging in such dangerous acts without fear of HIV/AIDS.

**Qn 3. What is your opinion on the burden of HIV among the youth (15-24 years) in this community?**

**Interviewee:** For me I see majority of the youth who are HIV positive are the ones who are born with it.

And according to my observation, majority of the youth who are HIV positive are the ones who are out of school because even those whom I know that are positive are those out of school.

**Probe on the commonest spread mode of HIV/AIDS**

**Interviewee:** For me what I think is that using sharp objects with those who are HIV positive is the commonest mode of spread because of poverty.

**Qn 4. What challenges do youth living with HIV/AIDS face?**

**Interviewee:** poverty is the biggest challenge faced by youth living with HIV/AIDS because most of them are enticed by old men just for the small money they give them and end up being infected by AIDS.

Another problem is stigma which makes even them even losing hope and school dropout thinking that there is no reason why they are studying yet they are going to die.

**Qn5.What solutions would you suggest tackling the above challenges?**

**Interviewee:** For me I think there should be a strong partnership between VHTS and schools so that we can educate the youth both at the school and out of school on how they should protect themselves from HIV/AIDS.

There should be enough education among the youth on how they should protect themselves from HIV/AIDS especially during holidays.

**Qun5.In what ways do you think youth in out of school get knowledge on HIV prevention strategies?**

**Interviewee:** For me I think they normally get information from the doctors and most radios are not good for educating youth on how to prevent themselves from HIV because majority of the youths don’t listen to such programs. They only like listening to Football and music.

Majority of the youths fear to approach us as VHTS because they say that we are too old than them. Therefore, they resort to going to health centers for more information.

**Probe on why some youths do not test for HIV/AIDS**

**Interviewee:** Majority of the youth fear to test for HIV/AIDS for the fear of being tested positive.

**Qn. What do you think are the challenges to adherence among the youth?**

**Interviewee:** Majority of the youth don’t take their medicine in time because they regard themselves are being strong. For example, out of 10 youths in my area who are positive,5 of them are the ones that adhere to the medicine.

**Probe on the perception of most youth with HIV/AIDS both at school and out of school**.

**Interviewee:** I think they are the youth who are in the school because those out of school are already married are no longer youths and others are not in the villages.

**Qn. How best do you think we improve access to the above HIV/AIDS care and treatment especially for the youth living with HIV/AIDS out of school?**

**Interviewee:** we should focus on mind set change towards positive thinking about living with HIV/AIDS. This can be done through community education on the dangers of living with HIV and also educating those with it on how they can live with it.

**Qn. Is there something you want to add to this interview, something we didn’t talk about but that you feel is important to you and want to share?**

**Interviewee:** I thank you for coming and also request that you always come in our villages and educate our youth especially in this period of holidays so that we as VHTS we mobilize our youth of you educate them on how they can prevent HIV/AIDS because they take HIV just for granted these days.

## INDEPTH INTERVIEW ONE_TEACHER (IDI T01)

**Qun. Can you tell me about yourself, about who you are and what you do at this school.**

**Interviewee:** My name is XXXX a teacher in charge of sanitation and health at XXXX school. And I teach Geography and Entrepreneurship.

**Qun. Can you describe a typical day in your life as a teacher in-charge of student’s health?**

**Interviewee:** it was last week when we had a visitor hear at school who normally bring matooke for the students, after delivering the matooke, the man tried to tour around and behind one these classroom blocks, there is a sewage that was not covered.so the man fallen into the sewage. And for me as the person concerned, I was shocked by such because I had no answer as to why the sewage was not covered.

For the HIV students, we have some students who are on ARVs, day our matron had a visit and she left me with the drugs. And that student had to take her drug at a half past nine, so I had gone to town after prep, and I remembered that that student had to take the tablets at when it was already five minutes passed time. So, I remembered that the student had to take the tablets and I was at the town, there were no transport means at that time, so I had to run and by the time I reached here, I found the student waiting for me at my door. And I reached here when I was too tired and tried to figure out what to tell the student. That was my typical day for me in handling students living with HIV.

And another day was that I was counselling students and I had to hit on he point that stigmatised one of the students living with HIV because sometimes you have to give information to all students so that they can fear HIV so I was wondering that should I take the student out, should I stop the communication, how best can I speak when this student is there? And the others keep the information. That was really a hard time for me.

**Qun. What is your opinion on the burden of HIV among the youth (15-24 years) in this community?**

**Interviewee:** As youth, when they acquire HIV, they become weak, they become hopeless in case they are not well counselled and that one reduces their productivity in terms of work.

**Probe on the number of youths living with HIV at his school**

**Interviewee:** At the moment, those which we are handling at the office they are two and also those handling themselves are also two so in total, we have four students and the most challenge to them is that they don’t want the school to know that they are HIV positive, so we also have to find the means of handling them.

**Probe on the different services available in schools as opposed to those out of school for the youth living with HIV**

**Interviewee**: Those ones in schools, they admit that they have it and they are taking their medication and they at least know that it is not the end of their life. However, those out of school it is very hard for them because they cannot motivate themselves and admit that they can live with it and even taking medication is becomes a problem for them.

**Probe on the services available for both out of schools and in the schools**

**Interviewee:** when it comes to the services available, to youth out schools, when they are tested positive, they become traumatized and fail to take medicine and immediately admit death, but services are there when they open up their mind because we have almost the health center three in each sub county and all services are available. Most of them think that contracting HIV may be is by joke and when they are tested positive, they take long to admit.

But those ones in schools they normally admit to treatment and know that there is life for them.

**Probe on the commonest mode of spread of HIV**

**Interviewee**: The commonest mode is through sexual intercourse and sharing sharp objects.

However, today we are recording high HIV prevalence because the youth has taken it normal to easily access sex even when they are not married because of relationships and even because of that anxiety, they don’t even ask their spouses about their HIV status.

**Qun. What challenges do youth living with HIV/AIDS in schools face?**

**Interviewee:** They most face a challenge of segregation because when the students know that they are HIV positive, they fear them and even they don’t what to share with them, even sharing uniforms. And from that, they feel lonely and fear to give out the problem they face to their friends and teachers.

**Qun. What solutions would you suggest tackling the above challenges?**

**Interviewee:** it’s to do sensitization to the fellow students on how to live with them so that they can be comfortable in the school.

**Probe on if they normally do sensitization at school on HIV**

**Interviewee:** No, we don’t do it but basically, we do it on other diseases but not on HIV because that of AIDS needs a trained councillor since us, we are used of punishing them, they may fear us to tell us most of the problems.

**Probe on comparation between what is being done and what is not being done and why in terms of HIV care, prevention, and treatment in his school**

**Interviewee:** currently, we are not taking the initiative to mind about these students in general like calling them and talking to them individually. We have just left them to live on their own ,they depend on the information they get from the health facility where they get the tablets and that one has left them to live in a very big distance towards their life and the school and the best solution to that is that maybe we would communicate with them first and also feel loved in the school .That one will comfort them so easily.

We have not done this before because in the school setting, we have the administrative structure and you cannot just weak up one day and implement a policy when you’re not among the policy makers even when you think of an idea that will help the student, you must report it to the person concerned.

**Qun. How do most of these youth in school get to know about their HIV status?**

**Interviewee:** At times, we do emphasis them to test for HIV when they go to the health facility, and we always recommend them to test for HIV on their permission cheats**.**

However, they have not been fully sensitized because they still take it for grated so me as a person in charge, this is a remainder to organize and talk to them to do some HIV testing.

**Qun. Tell me more about adherence to treatment among YLHIV in school?**

**Interviewee:** For the case of girls, the matron follows up for their adherence to treatment and for the case of boys, they are managing themselves.

**Qun. What do you think are the challenges to adherence among the youth?**

**Interviewee:** To the health centers where we normally refer to them, testing is free of charge however, the challenge they have is that they do not want to be identified. For them they think that when you cross to the HIV department, whether you’re sick or not! You will be identified as an HIV patient.so that what they fear but the testing is free. It is also challenge to them in terms of time management for drugs because some time they have to take them when its already time for classes, they need to rest after taking them and also eat well.

**Qun. What do you think are the challenges to adherence among the youth?**

**Interviewee:** May be those who take their drugs in the morning, can their routine and take them in after prep so that they have time for rest.

And for those taking them after prep, we need to find the food for them because they need to eat after taking them.

**Qun. How best do you think we improve access to the above HIV/AIDS care and treatment especially for the YLHIV in school?**

**Interviewee:** we should have a partnership with those government hospitals so that they come, and they have a healthy camp and provide HIV care, prevention, and treatment services to our students.

**Qun. What could facilitate better utilization of HIV/AIDS care, treatment, and prevention services for the youth out of school?**

**Interviewee:** I think there, they should use youth leaders in their community because they know themselves very well and prepare meetings and invite those medical workers to talk to them since most of the youth come from hard-to-reach areas that hinder them to get services.

**Qun. Is there something you want to add to this interview, something we didn’t talk about but that you feel is important to you and want to share?**

**Interviewee:** May be as researchers, on HIV care, prevention, and treatment services, you can make some flyers and posters about these awareness services so that we can put them in our schools because this also increases the awareness levels among the youth.

It would also be good if you have moved with a healthy person in charge of testing so that he/she can make a sample of some of our students to get a generalized sample from our school and also get the actual opinions from those youth willing and not willing to test.

**Qun. Are there any questions you have for the researcher?**

**Interviewee:** After this research, will you come back to do any impact because I believe that your research should be more impactful, or you will finish and publish and live things to remain like that?

## INDEPTH INTERVIEW TWO_TEACHER (IDI T02)

**QUN. Can you tell me about yourself, about who you are and what you do at this school?**

**Interviewee**: I am XXXX, a teacher by profession and posted here on a government payroll, I teach CRC and I am the school DOS as well as senior woman teacher.

**QUN. Can you describe a typical day in your life as a teacher in-charge of student’s health?**

**Interviewee:** For me I normally call them for guidance and counseling and that day I had called only girls as their senior woman teacher. I discussed with them and made them aware of HIV. I was using a piece of paper for everyone to talk about herself. A few confessed that they have it because they were born with it, and some didn’t know their status. And encouraged them to go and test and bring the results to me.

On that very discussion, some of them showed a lot of fear, they were worried, and, in the discussion, I was telling them that I am going to bring a health worker, and she will test us from here. And all of them become so worried. After, they went at the health facility and tested and two of them tested positive and as they brought the results, what I saw from them is that they were blaming each other that is themselves and their parents that they were not aware of HIV care, prevention, and treatment services.

**Qun. What is your opinion on the burden of HIV among the youth (15-24 years) in this community?**

**Interviewee:** The highest burden I see is that they cannot open up, it is confidential and when you observe, they cannot stop love affairs.my worry which is the burden to the school and community is that in the long run, they will end up infecting others.

And to me, some of them their parents do not know their HIV status. So it’s also a burden for us as teachers to approach their parents telling them that so and so is infected with HIV. This may even lead to most of them being paid school fees at school if they know that they are HIV positive because most of them come from uneducated families which see someone with HIV as useless people. They even have our Runyankole saying that ‘ogw’o afiire a yemerire’

Then another thing is that these people find it hard to go and get the drugs from the health centers because you know that there is no specific day for those school going youth and even no specific room for these age bracket and this becomes hard for the school going youth to line up with the elders and community members to get the ARVS.

According to my observation, they like free gifts from older people especially girls and it became high during this COVID 19 period and also the use of phones has also increased the spread because they use them to search phonography and you find that what they search, they want to practice them.

They are just you their eyes to judge who is or not positive and if they see someone healthy, they think that he/she is not HIV Positive.

They should also be taught on how they should abstain because during covid, most of them became a victim to HIV and others even have babies here, some were married and came back to school and a good number of them are on family planning. Their main concern is about pregnancy not HIV/AIDS.

**Probe on how many students living with HIV in her school**

**Interviewee**: yeah, they are there like eight of them.

**Qun. What challenges do youth living with HIV/AIDS in schools face?**

**Interviewee:** They don’t get the ARVS the way they should be. Then sometimes, they would need special food to boost their health. Another is that some of them are on and off, they miss lessons. These youths, they normally get angry very fast, and they are hard to handle since most of them are hopeless.

**Probe on the school dropout due to poverty and stigma to YLHIV**

**Interviewee:** we had one case of school dropout due to search cases and she got married. And for the issue of stigma, of course they are always there because there sometimes when your addressing students at the assembly and classes and you see such a student getting ashamed if you were telling her/him yet for you were addressing whole students about HIV care, prevention, and treatment services.

**Qun. What solutions would you suggest tackling the above challenges?**

**Interviewee:** continuous guidance and counseling is the only solution. Then may be if they can get support to get special food meant for them because I normally see people in the villages Some NGOS giving them water guards, packed food, and many others to support them .so if those are brought also in schools, that will be good.

Probe on what is being done compared to what is not being done to those YLHIV

Interviewee: we do counsel and guide, then also when they come for permission, we normally give it to them hence adhering to their request.

We would wish them to get medicine from here. Then get counsellors from outside because when the person is new, the message becomes different.

**Qun. In what ways do youth in school get knowledge on HIV prevention strategies?**

**Interviewee:** They normally get counseling services from outside and from us as teachers. We also normally put plays on TVs and watch what is going on. We also buy for them news papers and read what is going on.

**Qun. In what ways do you think youth out of school get knowledge on HIV prevention strategies**

**Interviewee:** some get information from their parents and friends. Then others get information from the person who is sick already because they see the person and say that according to what I see, he/she might be suffering from HIV/AIDS. Sometimes in our days we used to make drummer on HIV/AIDS because this passed information very well since it was made in local languages, but these days is not there because people pretend to be busy in other things [low voice and laughed].

**Qun. How do most of these youth in school get to know about their HIV status?**

**Interviewee:** It has been answered above

**Qun. Tell me more about adherence to treatment among YLHIV in school**

**Interviewee:** They do adhere well but that one comes after a long process of setting and counseling them well that they still have life.

**Qun. What would you suggest is the best way to tackle these challenges?**

**Interviewee:** The best way to me is that to have doctors and counsellors from outside and talk to them and then test them. And those who are negative and tell them the message about abstinence and to those who are positive, you also tell them messages that suits them like self-acceptance, abstinence so that they do not transmit it to others and show them life a head.

**Qun. How best do you think we improve access to the above HIV/AIDS care and treatment especially for the YLHIV in school?**

**Interviewee:** You know these things, need finance so to improve them means you should be equipped with money. But if everything is available, you would be making regular visits so that you know the challenges that we pass through and see how best can mobilize for us.

**Qun. Is there something you want to add to this interview, something we didn’t talk about but that you feel is important to you and want to share?**

**Interviewee:** we would wish to know how best those students YLHIV can come up and advocate, can stand by their own and say that they have HIVand tell others how they got it and how they are living with it. Because if they keep quiet, they will be more problems but if they come up, that one will save us more.

Then we have girls’ children here, they are from hamble families and if you can get some well wishers and give us at least sanitary pads because that’s also a big challenge here.

Then when they are taking ARVs, most of them hide from their parents meaning that there is a day when they miss because of that.so we need people who can sensitize their parents so that the parents aware of what their children are passing through so that they don’t create hatred on them.

**Qun. Are there any questions you have for the researcher?**

**Interviewee:** Should we have hopes that at one time we shall meet you again?

## INDEPTH INTERVIEW THREE_TEACHER (IDI T03)

**QUN.Can you tell me about yourself, about who you are and what you do at this school.**

**Interviewee**: I am called XXXX, I am a teacher here and I am a senior man.

**Qun. Can you describe a typical day in your life as a teacher in-charge of student’s health?**

**Interviewee:** I have ever had that day three years back but fortunately or unfortunately was a girl. That person was feeling too much stigma and she never wanted to be known when she was taking her drugs.so she could keep her drugs in her hostels and sometimes she could miss them and had many problems. But one day she become broadly and mentioned her problem to one of us and from there she was assisted, and her drugs were then kept in the office and every time she could come and take them freely.

**Qun. What is your opinion on the burden of HIV among the youth (15-24 years) in this community?**

**Interviewee:** unfortunately, I don’t have a clear record for the YLHV here because we have been disrupted by COVID19 and those whom we have been following have already completed.

The difference in service here is that we counsel our students who have got HIV from our senior men and women and when we are counseling them we normally do general counseling and also come to them directly for those YLHIV so they end up picking clear messages and go to health centers for example Rugaazi health center four and easily get medication from there with or with out us unlike those ones in the villages who have nobody to counsel them, talk to them and many others.

In fact, the main cause of HIV/AIDS among the youth is alcoholism and drug abuse because as these youth get out and get drunk, they find themselves indulging in sex with the victims of HIV/AIDS.so they just contact it out of unconsciousness due to the influence of drug abuse and alcoholism**.**

**Qun. What challenges do youth living with HIV/AIDS in schools face?**

**Interviewee:** Those students they are affected psychologically, physically they become weak, socially they feel that others may know that they have HIV/AIDS and become more stigmatized. So, there is a very big problem on their side.

They also lose hope and no future in them, and they even perform poorly because they see death very near so there is no reason to why they should be in school.

This could have contributed to the percentage of school droop out in this school because of the above challenges.

**Probe on how poverty is a challenge to those students**

**Interviewee:** yeah, having HIV is also expensive itself, they need good food, balanced diet, which we don’t provide to them here at school which needs money and if they are poor, there is no way on how they can get them which becomes a big challenge to them.

**Probe on how often they do HIV counselling at their school**

**Interviewee:** we normally do individual counselling to those students living with HIV/AIDS and for other students, we do guidance and counselling from different classes and age groups.

**Qun. What do you think are the challenges to adherence among the youth?**

**Interviewee:** From what I understand, those youth on drugs, they need a balanced diet, and they don’t find it here.

They would also need a nearby health facility where they would get the medicines from and remember those people giving out drugs have specific centers and many of them you find that they are registered from far and so get a challenge of going back to get the medicine. Even most of them do not adhere to the treatment because of those challenges.

**Qun. What would you suggest is the best way to tackle these challenges?**

**Interviewee:** The best way to tackle those challenges, let the ministry of health come out and be clear and make a policy that would really help the youth especially in schools to give them some help in terms of special food because in these schools, they do not find a balanced diet and yet they need to feed well.

And also let the schools be facilitated with such medicines for YLHIV or let the nearby health facilities be allowed to handle issues of learners for those YLHIV in terms of accessing the medicine there other than going back to the health centers where they were registered. So, if they find them, they could go to the nearby health center and then it would be okay, and we could be having a good message for them by directing them to those nearby health centers for good services but going back to the registered health centers is a big challenge to them.

**Qun. How best do you think we improve access to the above HIV/AIDS care and treatment especially for the YLHIV in school?**

**Interviewee:** like earlier said, let the ministry of health in corroboration with the ministry of education and sports at least register all learners in schools living with HIV/AIDS so that they get to know how many in the school like this one such that their medicine is brought to nearby health centers like Rugaazi health center four for easy accessibility.

**Qun. Is there something you want to add to this interview, something we didn’t talk about but that you feel is important to you and want to share?**

**Interviewee:** May be besides this oral interviewee, you could have left with me some questionnaire to fill for more detailed information than I could have done at this moment.

**Qun. Are there any questions you have for the researcher?**

**Interviewee:** I am okay with everything, and I don’t have any question to ask.

# CODEBOOK

Theme: Barriers to utilization of HIV prevention services

| **Category and codes** | **Quote** |
| --- | --- |
| Fear of testing: Fear of positive results, fear of stigma and discrimination, fear of starting life time treatment | - After reaching there ‘the room’, they did what they did…. then after two weeks from there, my friend started telling me that I was not sure whether this guy had HIV or not then later., after a month, she told me that she is not feeling okay. Then after we had to say. Do you know what you’re going to do you go at the hospital and test for HIV but besides she feared there[hospital] then we had to give her knowledge of going to the clinic and they tested her they found when, she is HIV positive (YIS FGD3 number7)      - I told her and I advised her that why can’t you go for testing she refused telling me that if you were you, you can fear. After I forced her and went with her in the hospital, and we found that she had an HIV. After she started…. she refused even take tablets and she ended up dying……… (YIS FGD2 number7)      - and also to find that they are HIV positive because there was one time when the youth asked me that ‘if you find that I am HIV positive, what would you do to me and I said that I will declare the results to you and she said that if you find that I am HIV positive, don’t give me the result slip that is positive. Give the result slip that is HIV negative because my parents are going to ask me’ so they are in that dilemma that when they are tested positive, it will be hard for them (IDI HW02)      - Most of the youth fear to test for HIV/AIDS thinking that if they are found with it, they are going to start taking tablets yet they fear the tablets for HIV/AIDS (YOS FGD2 number5)      - Some of the youth fear to test for the HIV/AIDS because most of them think that if they find them infected, they will be chased from their homes by their parents (YOS FGD2 number7) |
| Ignorance and misconceptions about HIV prevention services: lack of education on condom use, perceived reduction of sexual pleasure, fear of health consequences from condom use | - so that’s why I am saying that the use of condoms at some times its dangerous because you may find instantly or    carelessly and you find that it has gone into the vagina or the truck then at times your forced to go to the hospital to remove it or your find that your forced to get cancer (YIS FGD1 number8) - Even girls don’t want boys to use condoms when having sex for the fear that they don’t get sexual pleasure, that the boy may not put it on well and enters deep in their vagina and cause vaginal cancer. All the above prejudices can’t let them to use condoms which results into easy spread of HIV/AIDS among the youth (YOS FGD3 number6) - Then another thing of using condoms and whatever, also its dangerous though we emphasized to use them at times condoms you may use them hidden but not educated very well on how to use them most especially senior classes and primary level classes like P.7, P.6 and P.5(YIS FGD1 number8) |
| Low perceived susceptibility to HIV: lack of sensitization, overconfidence and denial | - The reason why some youth deep in the village don’t test for HIV is that some of the are very proud of themselves. They just say that me to acquire HIV! No…they just have that feeling (YIS FGD1 number1) - Majority of the youth take AIDS for granted and by the time they find that they have HIV, it rises stigma in them and fear to take tablets (YIS FGD2 number5) - However, they have not been fully sensitized because they still take it for grated so me as a person in charge, this is a remainder to organize and talk to them to do some HIV testing (IDI T02) |
| Lack of counselling services: absence of counselling services, limited access to trained counsellors, | - we don’t have any counseling services in our villages. Though if you find them at the nearby health centers, health workers can council you (YOS FGD3 number2) - For me I can’t lie, we don’t have those laws in our school and counseling services (YIS FGD2 number3) - we normally do general counseling and also come to them directly for those YLHIV so they end up picking clear messages and go to health centers for example Rugaazi health center four and easily get medication from there with or without us unlike those ones in the villages who have nobody to counsel them, talk to them and many others (IDI T01) - No, we don’t do it but basically, we do it on other diseases but not on HIV because that of AIDS needs a trained councilor since us, we are used of punishing them, they may fear us to tell us most of the problems (IDI T02) |
| Peer influence: discouragement from friends, dependence on the actions of peers | - Then another reason why some youths don’t go to test for HIV is because of peer group influence. You’re my friend then I ask you why you go to test for HIV? there is nothing long with you and you can’t go to test for HIV (YIS FGD1 number1) - Some youth wait to see others going to test for HIV/AIDS so that they can also go there. Therefore, if their fellows don’t go for HIV testing, they can’t go there as well (YOS FGD3 number2) |
| Stigma towards HIV: fear of being seen receiving treatment at the health facility | - Another thing is that stigma remains still a problem and because of this pandemic that have been there, it has put the youth on the high risk of acquiring HIV because there has been redundancy among youth and yet since they fear to be seen at the Health facility, they hide from there, the few who has come, are those who got pregnant or those who got married are the ones which access services and also because of the pandemic that happened (IDI HW02) |
| Lack of youth friendly services: limited access to HIV preventive services for youth out of school, absence of peer-based services | - And another thing is that most of our health facilities we don’t have youth friendly corners which means that we fail how to give youth services (IDI HW02) - Not at all because of two factors; one service and age go together because they also want their peer to serve them.so we are saying that it will be good if all health centers have a peer system especially office for the youth so that they know where they can meet their fellow youth and get services because it is still a general issue so there is still limited access of those youth out of school (KII 03) - Most of the youth fear to make lines in the hospitals with old people since there are no youth friendly corners which interferes their ability to test for HIV/AIDS (YOS FGD2 number8) |
| Breach of  confidentiality by health workers | - some youths don’t go to test for HIV because workers there at the health Centers when you go to test and you find that your HIV positive, they spread rumors that this one is positive, hence affecting you (YIS FGD1 number2) |

## Theme: Recommendations to utilization of HIV preventive services

| **Category and code** | **Quote** |
| --- | --- |
| Socioeconomic empowerment: need for income generating activities, need for skilling programs | - For those non-school going youth if they can be given income generating activities so that so that this system of going to the adult people yet they may be infected, so if they are given income generating activities, they may not go to seek for money from the older people (IDI HW02) - Economic empowerment if we can start some good programs that focus on youth like skilling. Yes, the youth could have been left school at an early age, but he/she have hands, she can go for saloon, carpentry, welding, and many others. So, they can be supported so that they can get something to survive on other than depending on support from the non-related parents (KII 03) |
| Continuous awareness campaigns: engagement of VHTs in community health education, collaborative efforts with schools, distribution of informational material, encouraging community health education | - You should always come in our villages and carry out civic education on how the youth should prevent HIV/AIDS and even us VHTs we can carry out role plays and songs regarding to the prevention of HIV/AIDS. we should always have youth meetings specifically on how they can prevent themselves from HIV (IDI VHT01) - I suggest that HIV education should be done at the village level because if it remains in the parish, majority of the youth don’t even go there and we should normally have new faces during that education because if they see us educating them, they don’t take it seriously because they are used to us (IDI VHT02) - For me I think there should be a strong partnership between VHTS and schools so that we can educate the youth both at the school and out of school on how they should protect themselves from HIV/AIDS (IDI VHT03) - May be as researchers, on HIV care, prevention, and treatment services, you can make some flyers and posters about these awareness services so that we can put them in our schools because this also increases the awareness levels among the youth (IDI T02) |
| Tracking | - we need to come up with contact tracing and identification of families that have HIV patients like them to some organizations that can help them (IDI HW03) - We should also encourage contact tracing and index testing for that family such that for any another positive can be known and put on treatment (IDI HW03) |
| Peer groups: Establishment of peer groups, utilization of youth leaders, mobile youth health workers | - We should also establish youth peer groups and they work with us at the facility and also work in the community because it is easy for a peer to encounter the youth at the village level (IDI HW02) - I think there, they should use youth leaders in their community because they know themselves very well and prepare meetings and invite those medical workers to talk to them since most of the youth come from hard-to-reach areas that hinder them to get services (IDI T02) - We should have community workers for the youth at the village level not the VHTS. we should have mobile youth health workers for the youth train them and give them what is necessarily like vehicles, Laptops, testing kits, CDS and move in the community to give services to the fellow youths in a humble way (KII 03) |
|  |  |

# Summary of themes, sub-themes and codes

| **Theme** | **Sub-theme** | **Codes** |
| --- | --- | --- |
| Barriers | Individual | Ignorance and misconception about HIV prevention services  Fear of testing  Low perceived susceptibility to HIV  Fear of breach of confidentiality |
|  | Community level | Lack of counselling services  Peer influence  Stigma towards HIV |
|  | Health system | Lack of youth friendly services |
| Recommendations | Community level | Formation of youth support groups  Socio-economic empowerment |
|  | Health system | Awareness campaigns |
