# Supplementary material for: “If you find that I am HIV positive, don’t tell me”: Exploring the barriers and recommendations for HIV prevention services utilization among youth in rural southwestern Uganda
Source: PLOS Glob Public Health. 2024 Sep 13;4(9):e0002555. doi: 10.1371/journal.pgph.0002555 (PMC11398690; doi:10.1371/journal.pgph.0002555)
Supplement: Attachment — Submitted filename: Response to Reviewers Comments.docx [file pgph.0002555.s002.docx]

**Response to Reviewers’ Comments**

| **Comment** | **Response** | **Line number** |
| --- | --- | --- |
| Major considerations |  |  |
| Were questions about PrEP included in these interviews? As one of the primary biomedical HIV prevention strategies, the absence of any content related to PrEP stands out as an important gap that was either not addressed in the interviews/focus groups or if asked, not included in the manuscript. This should be mentioned in the limitations section if it wasn’t included. | Thanks for bringing this to our attention. We acknowledge that PrEP is one of the primary biomedical HIV prevention strategies. Unfortunately, questions on PrEP were not included in the interviews and this has been acknowledged as a significant limitation. | Lines 481-485 |
| Results, recommendations section: Were youth participants asked about how to overcome barriers to accessing HIV prevention services? If so, did they corroborate any of the recommendations included? All of the quotations provided in the recommendations section were from key informants or in-depth interviews only (presumably all of whom are adults). The voices of the youth participants in focus groups would lend additional strength to the recommendations, since they are the primary focus of this study. | Thank you for your insightful comment. Indeed, as the primary focus group of this study, the youth participants were also consulted for potential recommendations on enhancing the utilization of HIV prevention services. Unfortunately, the feedback from the youth did not yield any significant recommendations that could be directly implemented. We attribute this to a general ignorance on existing HIV prevention services, and the low levels of literacy in this group.  Consequently, the study has relied more heavily on the perspectives of key informants and participants from in-depth interviews, who demonstrated a greater depth of knowledge on the subject. These contributions were considered significant and thus have been incorporated into the recommendations section. | N/A |
|  |  |  |
| Minor considerations |  |  |
| There are a number of grammatical or typographical errors throughout the manuscript (e.g., pg 4 line 97 “finishing villages” should read “fishing villages”). I didn’t highlight further examples here but would suggest a careful review of the manuscript to correct them. | Thank you for this insight, a thorough review of the manuscript has been made and all grammatical or typographical errors have been rectified. | N/A |
| Pg 4, lines 61-65: This sentence cites a reference (4) that details programming put in place in South Africa. Is there a different reference that specifically describes some of the HIV prevention strategies put in place in Uganda, which would be more relevant to the context of this study? | Thank you for highlighting this oversight regarding the contextual relevance of our citations. Upon your suggestion, we have identified and cited a more appropriate reference that specifically discusses the array of HIV prevention strategies implemented in Uganda. | Line 67-78 |
| Pg 4, lines 83-84: As best I can tell, this study doesn’t attempt to differentiate between barriers for youth in school vs. out of school. This sentence can probably be removed if that is the case. | Thank you for this comment. The statement has been retracted. | Lines 83-84 |
| Pg 4, lines 86-89: The objective of this study could use further clarification. The statement only describes exploring barriers to utilization of HIV prevention services and recommendations but doesn’t mention that this study is focused on barriers to HIV prevention services for rural youth in Uganda specifically. The opening sentence of the discussion section is more specific as to the study objective. | Thank you for noting this oversight. As mentioned in the opening statement of the discussion section, the main objective of this study was to explore barriers to the utilization of HIV prevention services among youth aged 15-24 years in rural southwestern Uganda and establish recommendations to improve the utilization of these services. The objective of the study in the lines 86-89 has been rectified to tally with the main objective. | Lines 85-89 |
| Pg 5, lines 112-115: I’m not especially familiar with protections for research participants in Uganda. Is it accepted practice that a head teacher can provide informed consent on behalf of a participant <18 years old in place of a parent or legal guardian? This doesn’t need to be added to the manuscript specifically. | Thank you for raising this concern. In this study, the requirement was for minors to have consent from a parent or legal guardian. However, due to logistical constraints that prevented the research team from obtaining consent from every minor's parent or legal guardian directly, head teachers were utilized as proxies for this purpose.  This approach was deliberated upon and subsequently approved by the institutional review board, ensuring that it aligned with ethical standards and was appropriate for the context of the study conducted in Uganda. | N/A |
| Pg 5, line 126: If youth participants and teachers were only selected from 4 schools in the district, it would appear that students and teachers from those 4 schools in the convenience sample were eligible, rather than all youth aged 15-24 in the district. Please clarify whether I’ve understood this correctly. | Thank you for your comment, which highlights a need for clarification regarding the recruitment of participants for our study. Indeed, the selection of youth participants and teachers from four schools might suggest a limited sample.  However, our recruitment strategy was inclusive of all youth aged 15-24 within the district, encompassing both those currently enrolled in school and those not attending school at the time of the study. The youth from the four selected schools served to represent the segment of the population that was in school. Additionally, to ensure a comprehensive inclusion of out-of-school youth, we employed a snowball sampling method facilitated by Village Health Team members. This approach allowed us to reach a broader spectrum of the youth population within the district, thereby enhancing the representativeness of our study sample. | Lines 115-126 |
| Table 1: could the authors clarify what the position “ART in-charge” means? While ART is an abbreviation I’m familiar with and that the authors have defined previously in the manuscript, the position name is still a bit confusing to me. Is this the person in charge of the ART program for a village? | Thank you for your inquiry. To clarify, "ART in-charge" refers to the individual responsible for overseeing the antiretroviral therapy (ART) program within a health facility in Uganda that provides ART services. This position involves managing the delivery of ART, ensuring the quality of care for patients receiving treatment, and coordinating ART-related activities within the facility. | N/A |
| Table 2: please provide column percentages for each cell. | Thanks for this suggestion. The column percentages have been included |  |
| Pg 11, lines 204-205: How the authors define barriers for the purpose of the study is probably more appropriate for the methods. | Thank you for your suggestion. We have revised the manuscript to relocate the definition of barriers, as utilized in this study, to the methods section. However, we have also chosen to retain a reference to this definition in the results section. This dual placement is intended to provide readers with immediate context when interpreting the findings, enhancing the coherence and accessibility of the discussion on barriers identified through the study. | Lines 94-95 |
| Discussion: In the introduction and methods, there was an emphasis on the fact that this study was done among rural youth in a district with many fishing villages (to emphasize the uniqueness of the context, I presume). However, the findings and discussion don’t really highlight whether the rural or fishing village context impacts the findings or whether any of the participants acknowledged that some of these barriers are in part because they live/work in a rural area (except for maybe the recommendation to expand awareness campaigns to the village level, pg 17 line 339-342). While not mandatory, contextualizing how some of the qualitative findings either compare to findings from urban settings or how delivering services in a rural setting might involve different barriers could be a useful addition where relevant. | Thank you for this feedback. We've updated the discussion to more clearly articulate the influence of the rural and fishing village setting on our findings. While direct participant acknowledgment of these contexts affecting barriers was not evident, their impact was inferred through some recommendations and barriers, such as the proposed need for economic empowerment to drive down the prevalence of transactional sex which is common in fishing communities. | Lines 440-447 and 456-467 |
