# Supplementary material for: “If you find that I am HIV positive, don’t tell me”: Exploring the barriers and recommendations for HIV prevention services utilization among youth in rural southwestern Uganda
Source: PLOS Glob Public Health. 2024 Sep 13;4(9):e0002555. doi: 10.1371/journal.pgph.0002555 (PMC11398690; doi:10.1371/journal.pgph.0002555)
Supplement: Attachment — Submitted filename: Response_to_Editor V3_16062024.docx [file pgph.0002555.s003.docx]

16th June 2024

Dear Editor PLOS GLOBAL PUBLIC HEALTH,

We extend thanks to you for providing comments to our manuscript. We have responded to the comments raised. We hope our manuscript is far better than it was before.

**Latest Comments 6/06/2024**

| 1. Please ensure that the Title in your manuscript and the Title in your online submission form are the same. | The title has been corrected. |
| --- | --- |
| 2. We notice that your supplementary table (Appendix) is included in the manuscript file. Please remove them and upload them with the file type 'Supporting Information'. Please ensure that each Supporting Information file has a legend listed in the manuscript after the references list. | The table has been removed from the manuscript and is uploaded as supporting information file 1 (S1). |
| 3. Please amend your detailed online Financial Disclosure statement. This is published with the article. It must therefore be completed in full sentences and contain the exact wording you wish to be published. a) State the initials, alongside each funding source, of each author to receive each grant. For example: "This work was supported by the National Institutes of Health (####### to AM; ###### to CJ) and the National Science Foundation (###### to AM)." b) State what role the funders took in the study. If the funders had no role in your study, please state: “The funders had no role in study design, data collection and analysis, decision to publish, or preparation of the manuscript.” | Financial Disclosure statement has been amended to “This work was jointly supported by the Fogarty International Center (U.S. Department of State’s Office of the U.S. Global AIDS Coordinator and Health Diplomacy [S/GAC] and the President’s Emergency Plan for AIDS Relief [PEPFAR]) of the National Institutes of Health (under Award Number R25TW011210 to CO) and Mbarara University of Science and Technology (MicroResearch grant 3/2022 to JKK). The funders had no role in study design, data collection and analysis, decision to publish, or preparation of the manuscript.” |
| 4. Please ensure that the funders and grant numbers match between the Financial Disclosure field and the Funding Information tab in your submission form. Note that the funders must be provided in the same order in both places as well. | This has been addressed. |
| 5. Please update your online Competing Interests statement. If you have no competing interests to declare, please state: “The authors have declared that no competing interests exist.” | This has been done. |

Earlier comments 15/05/2024

| **Editor’s comment** | **Response** |
| --- | --- |
| 1. Please indicate the theoretical model used in your study | We utilized the socio-ecological model in this study. This has been added to the manuscript lines 103-107 |
| 2. How did you decide on the number of FGDs and ID interviews to be conducted? | Following previous guidance, as cited on line 132, we planned for a minimum of five FGDs and five interviews, but discussions and interviews were held until information saturation. |
| 3. Please indicate the members of your team who were involved in the data collection and analysis in the methods section using their initials as well as what they brought to the study, | Thank you for pointing this out. This has been done across the methods section. |
| 4. Please note that in the qualitative analysis the codes cannot be directly abstracted into subthemes- they need to abstracted, and categorised. the categories need to be mapped under subthemes and themes. Hence, you seem to have missed some steps in the analysis. | Thank you for pointing out this. The subthemes and themes were deductively identified. The codes that were generated were abstracted into different categories, as presented in the results section. We have made this clearer in the data management and analysis sections, lines 175 to 177. |
| 5. Kindly address trustworthiness in your research- i.e., credibility, confirmability, dependability and transferability in terms of qualitative research. Also, please add a note on reflexivity within your study. | Thank you for identifying this oversight. The engagement with participants of different cadres and triangulation of data sources (FGDs, IDIs, KIIs) credibility and confirmability of our study findings. Additionally, to ensure transferability of our findings, a rich description of the research context is provided including the participants and their respective cadres, as well as the study setting. We have added this information to the discussion section, lines 480-490. |
| 6. Kindly work on categorising your codes and deriving subthemes and themes. The manuscript currently seems to have too many themes and would benefit from categorisation prior to arriving at these themes. Also, inference under each theme needs to be more comprehensive- the themes currently comprise quotes and very little inference. | Thank you for this comment. The analysis in this work followed both deductive and inductive approaches. The codes were inductively generated using thematic analysis, and subsequently categorized into three deductively generated sub-themes (following the socio-ecological model) and two main themes (barriers to and recommendations for the utilization of HIV preventive services). This allowed inference under each theme and sub-theme to be more comprehensive.  This process has been made clearer in the manuscript between lines 173-181. |
| 7. All quotes in the manuscript have a suffix- 03/2022, it is unclear what this means. You could clarify in the methods how you have denoted the quotes. | The suffix 03/2022 was used to indicate the month and year of the data collection for each quote, for easy traceability and context for the quotes provided. However, to ease readability, this has been removed. |
| 8. A table with examples of coding, abstraction, categorisation, arriving at subthemes and themes would be helpful. | Thank you for suggesting this. This table has been added to pages 55, 56 and 57 of the supplementary file 1. |
| **Reviewer’s comments** |  |
| 1.Pg 11, line 237: I believe there may be a typo or incomplete sentence “…barriers to utilization of HIV prevention services emerged during discussions and teachers with youth and teachers in charge of health schools.” | Thank you for bringing this to our attention. The typo has been corrected. |
| 2. Pg 18, line 405: sentence should begin with a capital letter. | This has been addressed. |

Sincerely,

Jerome Kahuma Kabakyenga

Corresponding Author
